# Robust Markers and Sample Sizes for Multicenter Trials of Huntington Disease

**DOI:** 10.1002/ana.25709

**Published:** 2020-03-14

**Authors:** Peter A. Wijeratne, Eileanoir B. Johnson, Arman Eshaghi, Leon Aksman, Sarah Gregory, Hans J. Johnson, Govinda R. Poudel, Amrita Mohan, Cristina Sampaio, Nellie Georgiou‐Karistianis, Jane S. Paulsen, Sarah J. Tabrizi, Rachael I. Scahill, the IMAGE‐HD, PREDICT‐HD, and TRACK‐HD investigators, Daniel C. Alexander

**Affiliations:** ^1^ Center for Medical Image Computing, Department of Computer Science University College London London United Kingdom; ^2^ Huntington's Disease Research Center, Department of Neurodegenerative Disease University College London, Queen Square Institute of Neurology London United Kingdom; ^3^ Queen Square Multiple Sclerosis Center, UCL Queen Square Institute of Neurology, Faculty of Brain Sciences University College London London United Kingdom; ^4^ Departments of Neurology and Psychiatry Carver College of Medicine, University of Iowa Iowa City IA; ^5^ Department of Biomedical Engineering College of Engineering, University of Iowa Iowa City IA; ^6^ Mary Mackillop Institute of Health Research, Australian Catholic University Melbourne Australia; ^7^ CHDI Management/CHDI Foundation New York NY; ^8^ Monash Institute of Cognitive and Clinical Neurosciences, School of Psychological Sciences, Faculty of Nursing, Medicine, and Health Sciences, Monash University Clayton Campus Victoria Australia

## Abstract

**Objective:**

The identification of sensitive biomarkers is essential to validate therapeutics for Huntington disease (HD). We directly compare structural imaging markers across the largest collective imaging HD dataset to identify a set of imaging markers robust to multicenter variation and to derive upper estimates on sample sizes for clinical trials in HD.

**Methods:**

We used 1 postprocessing pipeline to retrospectively analyze T1‐weighted magnetic resonance imaging (MRI) scans from 624 participants at 3 time points, from the PREDICT‐HD, TRACK‐HD, and IMAGE‐HD studies. We used mixed effects models to adjust regional brain volumes for covariates, calculate effect sizes, and simulate possible treatment effects in disease‐affected anatomical regions. We used our model to estimate the statistical power of possible treatment effects for anatomical regions and clinical markers.

**Results:**

We identified a set of common anatomical regions that have similarly large standardized effect sizes (>0.5) between healthy control and premanifest HD (PreHD) groups. These included subcortical, white matter, and cortical regions and nonventricular cerebrospinal fluid (CSF). We also observed a consistent spatial distribution of effect size by region across the whole brain. We found that multicenter studies were necessary to capture treatment effect variance; for a 20% treatment effect, power of >80% was achieved for the caudate (n = 661), pallidum (n = 687), and nonventricular CSF (n = 939), and, crucially, these imaging markers provided greater power than standard clinical markers.

**Interpretation:**

Our findings provide the first cross‐study validation of structural imaging markers in HD, supporting the use of these measurements as endpoints for both observational studies and clinical trials. **ANN NEUROL 2020;87:751–762**

The determination of robust and sensitive measures of disease stage is of increasing importance for Huntington disease (HD), where potential disease‐modifying treatments are maturing to the point of requiring large‐scale clinical trials.^1^ As a rare disease, it is essential that such trials in HD recruit participants from multiple centers to provide sufficient statistical power to detect treatment effects. Although imaging markers are seldom used as primary endpoints in clinical trials, they often provide important supporting information on the effects of treatment in neurodegeneration.^2^

Structural magnetic resonance imaging (sMRI) measurements of caudate and putamen volume perform particularly strongly as markers in HD, showing the earliest and greatest effect size for group differences and longitudinal change in both premanifest (PreHD) and manifest HD.^3^, ^4^, ^5^, ^6^, ^7^, ^8^, ^9^, ^10^ Other subcortical regions, such as the pallidum and accumbens, also undergo significant atrophy in HD gene carriers.^11^ However, atrophy in these regions is generally not identified as early as in the caudate and putamen, and it is unclear whether this is due to biological differences or measurement effects. White matter (WM) and cortical gray matter changes are also detected in HD.^6^, ^10^, ^12^ However, reported changes in cortical gray matter vary considerably.^13^, ^14^, ^15^, ^16^, ^17^, ^18^ We recently recapitulated the ordering of these observations—putamen and caudate before pallidum, then WM—using data‐driven computational disease progression modelling and sMRI data.^19^

Three of the largest cohort studies, PREDICT‐HD,^20^ TRACK‐HD,^10^ and IMAGE‐HD,^21^ were designed to undertake detailed characterizations of HD progression and to compare biomarker candidates. However, differences among enrollment criteria, study design, MRI acquisition, and processing pipelines mean that the results of previous work are difficult to compare and are often contrasting. Moreover, effect size and sample size estimations derived from sMRI measurements are highly dependent on the choice of processing pipelines.^22^

Here, for the first time, we retrospectively process and analyze imaging data from the PREDICT‐HD, TRACK‐HD, and IMAGE‐HD cohort studies to cross‐examine imaging markers in varying study designs and hence demonstrate their potential use in HD multicenter clinical trials. We use a single image processing pipeline to calculate volumetric measurements from sMRI data for all 3 cohorts. We use mixed effects models to adjust the volumetric measurements for covariates and hence compare adjusted measurements among cohorts to assess interstudy agreement. This approach allows us to identify a set of markers that show similar disease effects across all 3 studies and to compare the pattern of disease effects across the whole brain in a standardized manner.

Moreover, we use our data‐driven model to simulate hypothetical treatment effects and hence evaluate how the relative number of study centers and participants per center affects the statistical power of key markers. Crucially, our model suggests that imaging markers provide higher statistical power than standard clinical markers and that multiple centers are necessary to capture the variance of treatment effects. These findings have important implications for the use of imaging markers in future HD clinical trials and more broadly on the design of clinical trials for rare diseases.

## Subjects and Methods

### *Cohorts*

Participants from the PREDICT‐HD, TRACK‐HD, and IMAGE‐HD studies with measurements at 3 time points (study baseline plus 2 follow‐ups) were included in all analyses. Additional follow‐up data were available from the PREDICT‐HD and TRACK‐HD studies, but to reduce sampling bias the number of follow‐ups was made consistent across all 3 studies. Furthermore, to minimize confounds of intrasubject time‐dependent measurement effects, participants were required to have measurements from the same scanner and field strength at all 3 time points. Finally, participants had to pass a visual quality control (QC) on each brain scan and segmentation at each time point (see Statistical Analysis section for details). Using these criteria, we selected 265 participants from 20 centers in PREDICT‐HD, 294 participants from 4 centers in TRACK‐HD, and 65 participants from 1 center in IMAGE‐HD (see Supplementary Table 2 for the number of participants at each stage of selection). We note that no participants underwent any disease modifying treatment during data collection.

#### *PREDICT‐HD*

Participants were recruited at 33 global centers, with nearly all participants recruited to be PreHD or healthy controls (HCs).^20^ All participants were required to undergo genetic testing (cytosine, adenine, guanine [CAG] of ≥39 repeats) independent of the research study. PREDICT‐HD had rolling enrollment between 2001 and 2012, with a total of 1,013 PreHD and 301 gene‐negative controls recruited. Participants were diagnosed according to standard diagnostic criteria requiring a score of 4 on the Unified HD Rating Scale (UHDRS) diagnostic confidence level (DCL),^23^ meaning that the participant has  motor abnormalities at a level representing unequivocal signs of HD. Participants were excluded from the study at enrollment if there was a diagnosis of HD or evidence of an unstable illness, alcohol or drug abuse, a history of special education or central nervous system disease, a pacemaker or metallic implants, antipsychotic medications prescribed in the previous 6 months, or use of phenothiazine‐derivative antiemetic medication for 3 months or more. Acquisition parameters for the PREDICT‐HD scanners included in this analysis are provided in Supplementary Table 1. Study activities were reviewed and approved by institutional review boards at all study and data processing sites. Participants underwent informed consent procedures and signed consents for both participation and to allow deidentified research data to be sent to collaborative institutions for analysis.

#### *TRACK‐HD*

Data for TRACK‐HD were collected at 4 centers: Leiden, London, Paris, and Vancouver.^10^ HD gene carriers were required to have a CAG of ≥40 and were recruited from clinics at each center. At baseline, 123 controls were recruited, along with 120 PreHD participants and 123 HD participants. PreHD participants were required to have a burden of pathology score of >250 (indicating that they are close to onset, calculated as [age × (CAG − 35.5)])^24^ and a UHDRS Total Motor Score (UHDRS‐TMS) of <5, indicating minor motor symptoms.^25^ Manifest HD participants were required to have a DCL of 4 and a Total Functional Capacity (TFC) of 7 or more, as measured by the UHDRS‐TFC.^23^, ^24^ 3T T1‐weighted scans were acquired from 4 scanners (2 Siemens [Erlangen, Germany], 2 Philips [Amsterdam, The Netherlands]). The parameters for Siemens were repetition time (TR) = 2,200 milliseconds, echo time (TE) = 2.2 milliseconds, field of view (FOV) = 28cm, matrix size = 256 × 256,208. For Philips, TR = 7.7 milliseconds, TE = 3.5 milliseconds, FOV = 24cm, matrix size = 242 × 224,164. The acquisition was sagittal to cover the whole brain. There was a slice thickness of 1mm, with no gap between slices. These acquisition protocols were validated for multisite use.^10^ The study was approved by the local ethics committees, and written informed consent was obtained from each participant.

#### *IMAGE‐HD*

IMAGE‐HD was a single‐center study with control, PreHD, and manifest HD participants.^21^ Gene carriers had a CAG of ≥39 repeats, and PreHD and manifest HD participants were allocated to each group based on their UHDRS‐TMS, with those having a score of 5 or less included in the PreHD group and participants with a score of greater than 5 included in the manifest HD group. There were 108 participants recruited at baseline, with imaging data available for 31 PreHD, 31 manifest HD, and 29 control participants. Data were collected using a Siemens Magnetom Tim Trio 3T scanner with a 32‐channel head coil. *T*1‐weighted images were acquired with 192 slices, 0.9mm slice thickness, 0.8mm × 0.8mm in‐plane resolution, TE = 2.59 milliseconds, TR = 1,900 milliseconds, and flip angle = 9°. The study was approved by the Monash University and Melbourne Health Human Research Ethics Committees, and informed written consent was obtained from each participant prior to testing in accord with the Helsinki Declaration.

### *Other Variables*

To facilitate comparison among the 3 studies, 3 measures of clinical progression were quantified for each cohort: UHDRS‐TMS, DCL, and UHDRS‐TFC, although this was not available for the IMAGE‐HD study. Two cognitive scores from the UHDRS—the symbol digit modalities test (SDMT)^26^ and Stroop word reading test (SWRT)^27^ —were used as cognitive outcome measures, the UHDRS‐TMS was used as motor outcome measure, and the disease burden score (DBS)^28^ was used to quantify approximate lifetime disease burden.

### *Image Analysis*

Structural MRIs from a total of 265 PREDICT‐HD, 294 TRACK‐HD, and 65 IMAGE‐HD participants at baseline plus 2 follow‐ups were analyzed. T1‐weighted MRI data at 3T were used from the TRACK‐HD and IMAGE‐HD datasets and at 1.5T (n = 215) and 3T (n = 50) from the PREDICT‐HD dataset. Table 1 shows demographic data from each study at baseline, and Supplementary Tables 3 and 4 show similar tables for each follow‐up.

**Table 1:** Baseline Demographics of Participants

Demographic Characteristics	Control Participants			PreHD Participants			HD Participants		
	PREDICT‐HD	TRACK‐HD	IMAGE‐HD	PREDICT‐HD	TRACK‐HD	IMAGE‐HD	PREDICT‐HD	TRACK‐HD	IMAGE‐HD
n	56	106	23	205	105	22	4	83	20
Age	45.1 ± 12.1	46.3 ± 10.2	44.4 ± 13.9	41.8 ± 10.8	41.1 ± 8.8	43.4 ± 8.3	46.8 ± 10.7	49.1 ± 9.5	53.4 ± 8.8
Sex, male:female	36:20	61:45	16:7	129:76	56:49	16:6	3:1	44:39	7:13
TIV	1.37 ± 0.134	1.39 ± 0.133	1.44 ± 0.144	1.37 ± 0.13	1.4 ± 0.146	1.34 ± 0.141	1.32 ± 0.128	1.37 ± 0.124	1.41 ± 0.156
CAG	20.3 ± 3.3	NA	NA	42.3 ± 2.6	43.0 ± 2.3	42.0 ± 2	43.0 ± 4.2	43.6 ± 3.1	42.9 ± 2.4
TMS	3.3 ± 3.8	1.6 ± 1.7	NA	5.2 ± 5	2.6 ± 1.7	1.0 ± 1.3	21.2 ± 13	23.2 ± 10.9	18.2 ± 9.4
DCL	0.5 ± 0.8	0.3 ± 0.4	NA	0.9 ± 0.8	0.6 ± 0.6	NA	4.0 ± 0.1	4.0 ± 0.1	NA
TFC	13 ± 0.1	12.4 ± 1.3	NA	12.6 ± 1.8	12.2 ± 1.6	NA	12 ± 1.4	12.2 ± 1.6	NA
DBS	NA	NA	NA	267.1 ± 71.8	293 ± 47.7	272.1 ± 55.4	320.7 ± 114	374.7 ± 77.7	377.1 ± 74.2

Demographic data for the PREDICT‐HD, TRACK‐HD, and IMAGE‐HD participants at baseline.

CAG = cytosine, adenine, guanine; DBS = disease burden score (CAG × years); DCL = diagnostic confidence limit; HD = Huntington disease; NA = not available; PreHD = premanifest Huntington disease; TFC = total functional capacity; TIV = total intracranial volume; TMS = total motor score.

MRI data were provided by data controllers in Neuroimaging Informatics Technology Initiative (NIfTI) format and were postprocessed to acquire regional measurements of brain volumes using the Geodesic Information Flow (GIF) software framework.^29^ GIF was designed to perform regional segmentation of T1 scans and was evaluated on scans of severe Alzheimer pathology, where it performed significantly better than other automated tools when compared to manual segmentation.^29^ It produces 156 regional brain volumes, corresponding to the brain atlas (Neuromorphometrics, Somerville, MA), and includes correction for intensity bias within the images. Here, because previous work has shown few hemispheric differences in HD atrophy,^12^ we combined bilateral volumes to give a total of 83 regional volumes. Total intracranial volume (TIV) was output from the GIF segmentation and was calculated as the sum of cerebrospinal fluid (CSF), cortical gray matter, deep gray matter, and WM.

**Figure 1:**
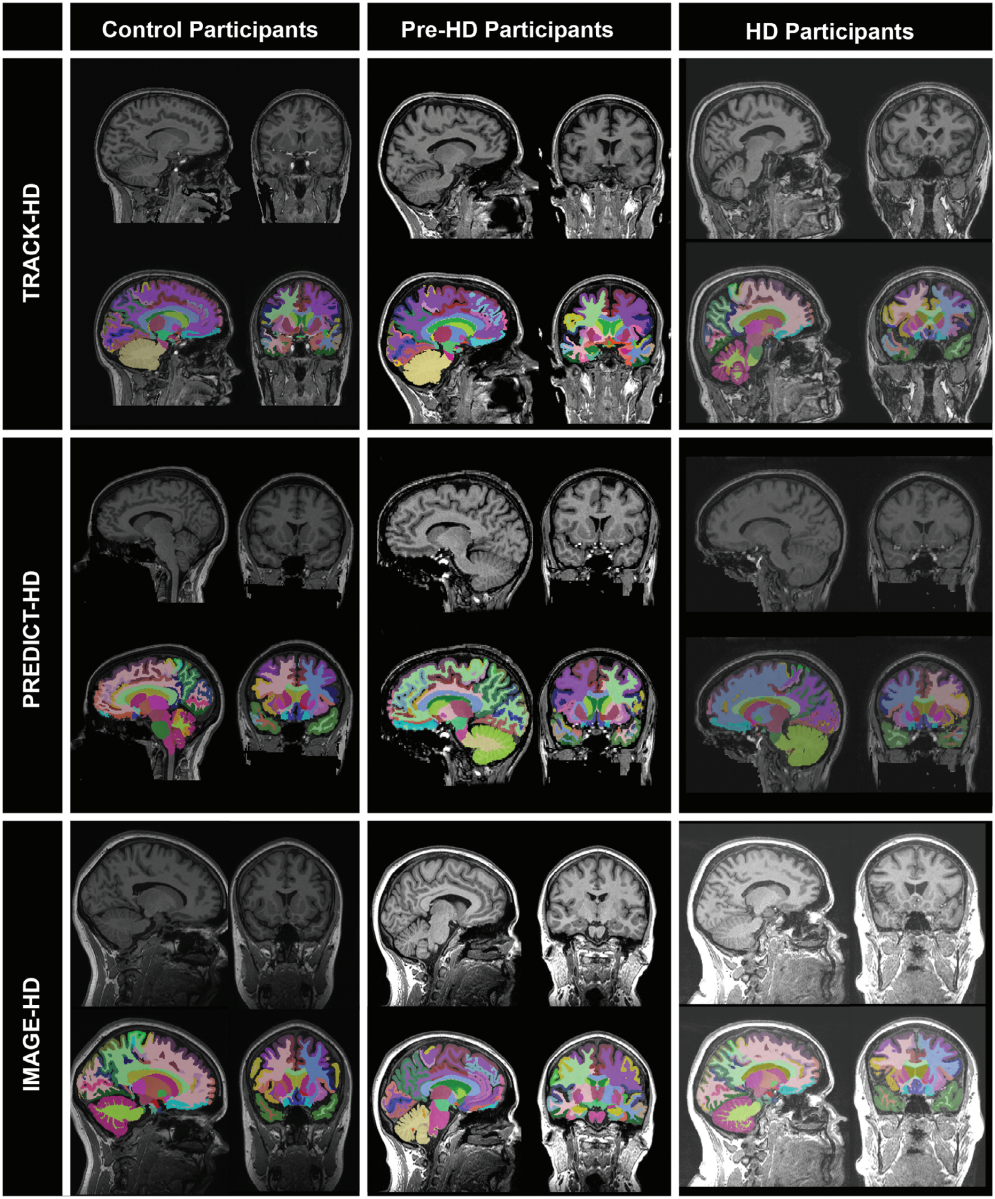
Randomly selected examples of T1‐weighted scans and T1‐weighted scans with GIF segmentation overlays for TRACK‐HD (top row), PREDICT‐HD (middle row), and IMAGE‐HD (bottom row) for controls (left column), premanifest Huntington disease (PreHD; middle column), and manifest HD (right column).

Imaging data from all cohorts underwent visual QC to check for issues in the data collection or processing. Scans were qualitatively checked by a trained reviewer for artefacts such as significant motion or poor FOV positioning, and the segmentations were checked for abject failures. No quantitative criteria were used for the QC process. Within the PREDICT‐HD dataset, the most common problem was due to the defacing procedure. Defacing aims to maintain anonymity by wiping facial features from MRI scans, and the data were provided with this procedure already performed; however, in some scans the frontal lobes were wiped along with facial features, producing inaccurate segmentations. Furthermore, in a small number of cases the segmentation excluded regions of gray matter from the frontal lobes, and these were removed from the analysis. No other consistent issues were seen with the segmentation or the data quality, although data quality was variable. The most substantial variation in data quality was seen in the PREDICT‐HD study, which is to be expected given that it also has the largest number of sites. After QC was performed, 36 participants from the PREDICT‐HD cohort and 2 participants from the IMAGE‐HD cohort were found to have considerable movement artefacts, defacing issues, or poor segmentations and were removed from the analysis. Figure 1 shows examples of scans and associated segmentations to demonstrate the data quality for this study. The top panel shows a TRACK‐HD PreHD participant where there is minor spillage of the pallidum and caudate segmentation beyond the anatomical boundary.

### *Statistical Analysis*

#### *Comparison of Adjusted Volumes*

To adjust regional volumes for covariates and estimate their rates of change, we used linear mixed effects models with the regional volume as the dependent variable and time and covariates as fixed effects.^30^ As such, the adjusted regional volume at baseline was given by the model at t = 0. Participants were nested in center as a random effect on the intercept. Covariates included as fixed effects were time, age, sex, TIV, and group (PreHD vs control) and an interaction term between time and age. In addition, the TRACK‐HD 3T data included scanner as a fixed effect, and the PREDICT‐HD 1.5T + 3T data included both scanner and voxel size as a fixed effect. Study was considered as an additional fixed effect but encoded the same information as scanner in the IMAGE‐HD 3T cohort, and hence scanner was used as it was more informative.

#### *Effect Sizes between Control and HD Groups*

Standardized effect sizes were estimated between HC and PreHD participants at baseline for each adjusted volume separately. Unless otherwise stated, *p* values were Bonferroni adjusted for multiple comparisons. To facilitate direct comparison among the 3 studies, standardized effect sizes were calculated as the difference in means between the HC and PreHD groups divided by the residual standard deviation in the PreHD group.

#### *Prediction of Treatment Effect*

The power of a hypothesized treatment effect for a given regional volume was estimated by simulation.^31^ The covariate‐adjusted hyperparameters estimated from the mixed effects model were used to inform the statistical power model and hence estimate power as a function of the nested variables (participants nested in center).^32^ The R statistical software framework^33^ with the LMER package^34^ was used to fit all models and simulate data. Open‐source code is provided by the authors at: https://github.com/pawij/lme_model.

## Results

Demographics are reported in Table 1, with *p* values between each pair of studies reported in Supplementary Table 5. We note that the 3 studies were optimized for observing different subcohorts of the HD spectrum, and hence study‐specific inclusion criteria influenced cohort differences among studies. Most notably, UHDRS‐TMS was significantly different among all 3 cohorts, and TRACK‐HD participants had significantly higher mean CAG length and DBS (*p* ≤ 0.001) compared to PREDICT‐HD.

The influence of different inclusion criteria for each study group was tested by including Study × Group as an interaction term in the mixed effects model. Tests were performed between the mean group value and the interaction between study and the group of interest (either PreHD or manifest HD) and Bonferroni‐adjusted for multiple comparisons. Differences were noted between HD groups in PREDICT‐HD and TRACK for the pallidum (*p* < 0.0001) and amygdala (*p* < 0.05) and between HD groups in TRACK‐HD and IMAGE‐HD for the caudate (*p* < 0.05). No significant differences were observed between PreHD groups.

To compare regional volumes among studies, numerical values for each region are provided in Supplementary Tables 6 and 7, which show raw and adjusted volume data, respectively.

### *Largest Effect Sizes Are Consistent among Studies*

**Figure 2:**
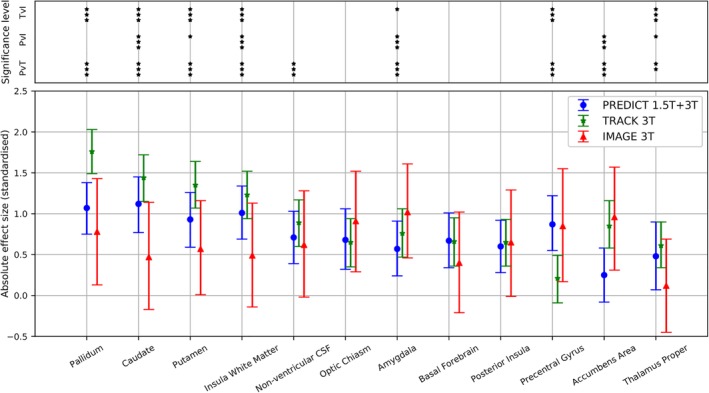
Absolute value of the standardized effect size between control and premanifest Huntington disease (PreHD) groups for regional volumes with a weighted mean of >0.5 in the PREDICT 1.5T + 3T, TRACK 3T, and IMAGE 3T cohorts. The 95% confidence intervals from 2,000 bootstraps of the data are shown. Weights are proportional to the total number of participants in each cohort (control + PreHD). Regions are ordered by the weighted mean across the 3 cohorts. The bar at the top denotes significance levels between each pair of studies: PREDICT‐HD versus TRACK‐HD (PvT), PREDICT‐HD versus IMAGE‐HD (PvI), TRACK‐HD versus IMAGE‐HD (TvI). ***p < 0.0001; **p < 0.001; *p < 0.05. All *p* values are Bonferroni adjusted for multiple comparisons. CSF = cerebrospinal fluid. [Color figure can be viewed at http://www.annalsofneurology.org]

Figure 2 shows standardized effect sizes, t, between HC and PreHD groups at baseline, for volumes with a weighted absolute mean standardized effect size |t| > 0.5, separately in the PREDICT‐HD, TRACK‐HD, and IMAGE‐HD cohorts. The weighted mean was calculated using the total number of participants in the HC and PreHD groups. Confidence intervals were estimated at the 95% level by bootstrapping, using 2,000 replications. The |t| > 0.5 threshold is semiarbitrary and follows the rule of thumb proposed by Cohen to identify small (0.2), medium (0.5), and large (0.8) effect sizes.^35^ This selection criterion identified 12 regional volumes, in descending order of |t|: pallidum, caudate, putamen, insula WM, nonventricular CSF, optic chiasm, amygdala, basal forebrain, posterior insula, precentral gyrus, accumbens area, and thalamus proper.

There was general consistency among studies, except in the first 4 regions (pallidum, caudate, putamen, insula WM), which had significantly larger effect sizes in TRACK‐HD 3T than in PREDICT‐HD 1.5T + 3T or IMAGE‐HD 3T (*p* < 0.0001). The larger effect sizes in TRACK‐HD were likely due to the PreHD group having a higher mean DBS than either PREDICT‐HD or IMAGE‐HD PreHD groups (see Table 1). See also Supplementary Table 8 for the complete set of effect sizes for all 83 volumes. We noted that the regions following the top 12 were also disease‐related (motor and frontal) and that these regions clustered together in effect size magnitude.

### *Pattern of Effect Sizes Consistent among Studies*

**Figure 3:**
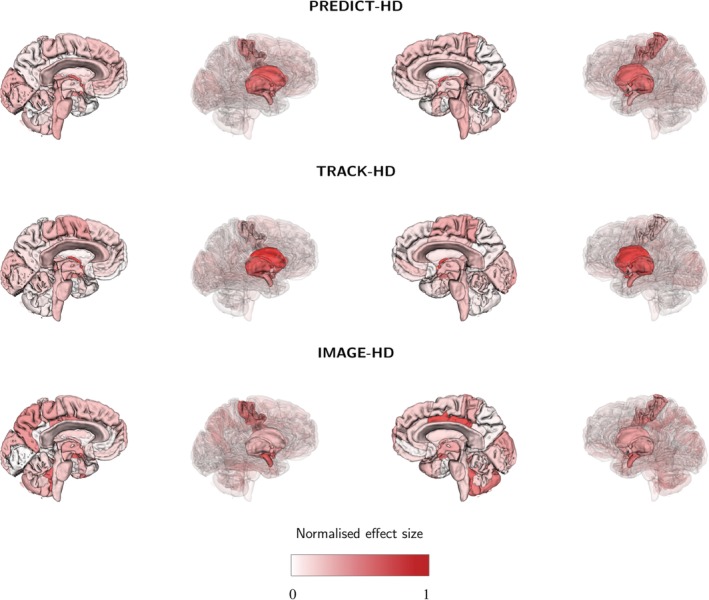
Standardized effect size between healthy controls and premanifest Huntington disease (PreHD) groups for each regional volume and for each study. For visualization purposes, the effect size shown is normalized to the largest effect size across all studies, which was observed in the right pallidum in TRACK‐HD (|t| = 1.74).

Figure 3 shows a graphical representation of effect size by region across both hemispheres of a model brain. For visualization purposes, the effect size was normalized to the maximum effect size across all 3 studies, which was observed in the right pallidum in TRACK‐HD (|t| = 1.74). General agreement was observed among studies, with the largest effect sizes found in the striatum and the largest magnitudes observed in TRACK‐HD. Again, this was expected due to the higher mean DBS in TRACK‐HD. Notably, the pattern of effect sizes was visually more similar between PREDICT‐HD and TRACK‐HD than between either cohort and IMAGE‐HD. This finding reflects the closer agreement in adjusted volumes between PREDICT‐HD and TRACK‐HD (see Supplementary Table 7) and the noisier signal from IMAGE‐HD due to small sample size.

### *Multicenter Imaging Markers Provide Greater Statistical Power than Clinical Markers*

We used the mixed effects model trained on data from all 3 studies to estimate the statistical power of the pallidum, caudate, putamen, insula WM, and nonventricular CSF. This corresponded to the top 5 regions with standardized effect size |t| > 0.5 in all 3 studies, that is, regions with a medium effect size in each study separately. Three years of observational data were used to inform the model, corresponding to baseline measurements and 2 follow‐ups. Participants from both the PreHD and manifest HD groups of all 3 studies were used, to best reflect the conditions of a clinical trial.

**Figure 4:**
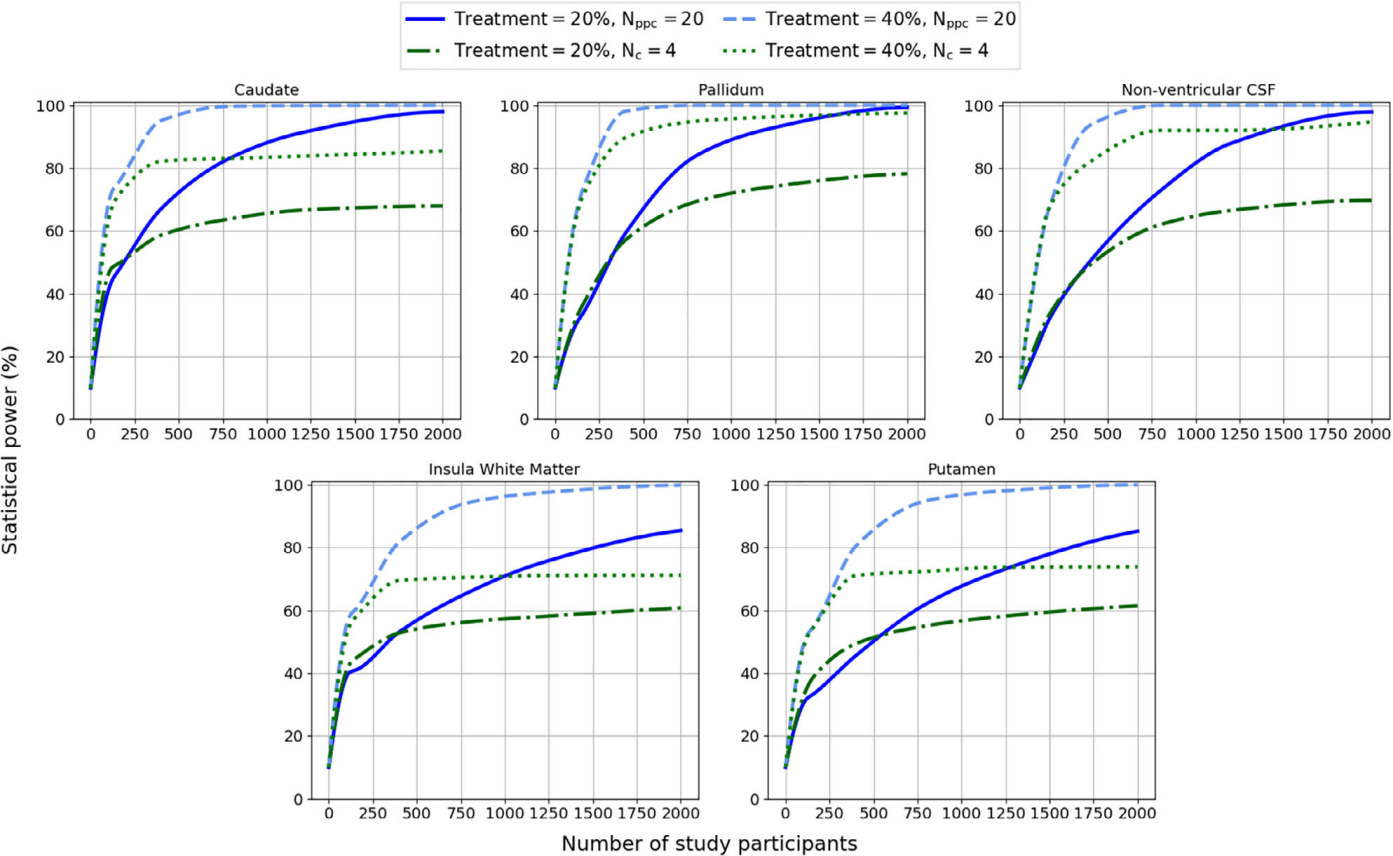
Simulation results estimating statistical power as a function of total number of study participants, for varying number of centers (Nc) and fixed number of participants per center (Nppc; Nppc = 20) and for varying Npcc and fixed Nc (Nc = 4). Hyperparameters (intercept, effect size, and variances) were estimated directly from longitudinal PREDICT‐HD 1.5T + 3T, TRACK‐HD 3T, and IMAGEHD 3T data (baseline +2 follow‐ups). Treatment effect significance was assumed under a 2‐tailed *t* test, with α = 0.05. The number of time points was fixed at 3 for all simulations. CSF = cerebrospinal fluid. [Color figure can be viewed at http://www.annalsofneurology.org]

Figure 4 shows power as a function of the total number of study participants for treatment effects of 20% and 40%. Table 2 shows predicted values for the total number of study participants necessary to reach a threshold power of >80% for each of the 5 volumes; we adopted the convention that power > 80% is considered to have rejected type II error,^36^ with significance assumed under a 2‐tailed *t* test (*α* = 0.05).

**Table 2:** Statistical Power of Selected Regional Volumes

Region	Treatment Effect, %	Number of Centers	Number of PPTS per Center	Threshold PPTS (Power > 80%)^a^
Caudate	20	(6–100)	20	661
	20	4	(30–500)	—
	40	(6–100)	20	200
	40	4	(30–500)	286
Pallidum	20	(6–100)	20	687
	20	4	(30–500)	—
	40	(6–100)	20	198
	40	4	(30–500)	230
Nonventricular CSF	20	(6–100)	20	939
	20	4	(30–500)	—
	40	(6–100)	20	242
	40	4	(30–500)	326
Insula white matter	20	(6–100)	20	1,445
	20	4	(30–500)	—
	40	(6–100)	20	358
	40	4	(30–500)	—
Putamen	20	(6–100)	20	1,560
	20	4	(30–500)	—
	40	(6–100)	20	378
	40	4	(30–500)	—

Number of participants necessary to achieve statistical power > 80%, for each volume, for various treatment effects and numbers of centers and participants per center. Inclusive ranges of number of either centers or participants are shown in parentheses.

a In cells without data, the threshold was not reached before the maximum total number of participants (N = 2,000). CSF = cerebrospinal fluid; PPTS = participants.

In both the variable‐center and variable‐participants‐per‐center tests, the highest powers were obtained in the caudate and pallidum with a variable number of centers. Fixing the number of centers and varying the number of participants per center achieved threshold power only in the caudate, pallidum, and nonventricular CSF for treatment effect of 40%. The reduction in power when fixing the number of centers was expected because the treatment effect variance was nonzero and hence a large number of centers was necessary to capture the variance.

To compare with a standard outcome measure for clinical trials, we also fit a mixed effects model to UHDRS measures TMS, SDMT, and SWRT from the same dataset. Here age, sex, and group were included as fixed covariates, and center and participant nested in center as random effects. We found that TMS only achieved powers of approximately 42%, SDMT 29%, and SWRT 25% for up to 2,000 total study participants with a 20% treatment effect. This was due to TMS, SDMT, and SWRT being highly variable markers of disease progression,^25^, ^37^ that showed only a weak dependency on time with the model used here.

## Discussion

For the first time we have performed a retrospective analysis comparing structural imaging data using 3 large HD cohorts (PREDICT‐HD, TRACK‐HD, and IMAGE‐HD) to determine robust imaging markers and derive upper sample size estimates for HD clinical trials. Importantly, these 3 cohorts offer a unique opportunity to draw inferences about sMRI markers and to determine their capabilities as clinical trial endpoints, from small phase 1 trials to much larger phase 3 trials. This has particular relevance to HD, which is rare and thus requires multicenter data, even at phase 1.

We identified a common set of regions that had consistently large effect sizes across multiple studies (see Fig 2). Subcortical regions, such as caudate and putamen, are well‐established imaging markers in HD, and here they had consistently large effect sizes across all 3 studies. In addition, the pallidum, nonventricular CSF, and the amygdala showed large effect sizes. These regions have previously been reported to differ between controls and PreHD yet are often not included in analyses, partly due to the difficulty associated with segmenting these structures.^7^, ^38^, ^39^, ^40^ Although the absolute value of the effect sizes for the set of regions shown in Figure 2 varies among cohorts, these regions consistently show effect size >0.5 and are the most congruous markers across 3 studies with very different designs. Moreover, within each study the pattern of effect sizes was similarly distributed across the brain (see Fig 3). Again, the magnitudes vary among cohorts, due to the aforementioned effects of sample size, sample noise, and disease stage of HD gene carriers.

The power analysis performed here uniquely uses data from 3 large cohorts to validate sMRI markers, with the caudate, pallidum, putamen, insula WM, and nonventricular CSF being compared. Combining the 3 cohorts provides a broader range of data than any 1 study alone and is therefore representative of multicenter trials with variable participants, scanners, and clinical assessors. To achieve statistical power of >80% for a 20% treatment effect, the caudate required the fewest number of participants (n = 661), and the pallidum required only slightly more participants (n = 687). Volumetric measures of the caudate, putamen, and ventricles have been used in clinical trials (https://clinicaltrials.gov/ct2/show/NCT02215616), yet the results presented in Figure 4 suggest that much higher numbers of participants may be required to detect a significant effect of medication on regions other than the caudate, pallidum, or nonventricular CSF and that many centers are necessary to capture intercenter variability. We note that these estimates are an upper bound, as the cross‐study variation present in the data used here should be higher than that for a prospective multicenter clinical trial. Furthermore, we do not explore the potential of composite endpoints, for example, that combine caudate and pallidum volumes into a single marker. Additional simulations (not shown) suggest that such measures could increase power further, but we leave detailed exploration of this idea to future work.

The PREDICT‐HD study was the first large observational MRI study to commence in HD and included a wide range of centers, scanner types, and field strengths to recruit over 1,000 participants globally. This study reflects the design of many clinical trials that include imaging, with 1 recent clinical trial in HD recruiting 352 participants at 52 centers (https://clinicaltrials.gov/ct2/show/NCT02215616). Thus, as in a clinical trial, the PREDICT‐HD data are somewhat noisier than in the TRACK‐HD study, which recruited participants at 4 centers. The noise introduced by a large number of centers is counterbalanced by the large sample size that can be recruited to a study with more centers, and the analysis performed here indicates that the increased noise and even the inclusion of multiple field strengths does not result in a detrimental reduction in effect sizes compared to the 2 other studies. Conversely, despite having a small sample size, IMAGE‐HD was able to identify the same set of regions as the other 2 studies. This type of cross‐study retrospective evaluation is essential to rectify the differing conclusions frequently reached when studying brain differences in sMRI analyses.

It is interesting to note that the regions with the largest effect sizes differed among studies; in PREDICT‐HD it was the caudate, in TRACK‐HD the pallidum, and IMAGE‐HD the middle cingulate gyrus. These findings support previous results that have shown differing disease effects across brain regions^2^ and suggest that the difference may be due to inconstant inclusion criteria, which results in the mean disease stage varying between studies. This suggests that different markers may be appropriate for different disease stages. As with motor and cognitive tests that show time‐dependent sensitivity at various stages of HD progression, the most appropriate imaging markers for a given trial or study may depend on the disease stage of interest. More sophisticated analysis techniques that combine information from multiple regions and account for disease stage, such as disease progression modelling,^41^ present an opportunity to derive integrated markers for fine‐grained cohort stratification.

It is important to recognize caveats in this analysis. In large cohorts, automated tools enable the delineation of many regions and provide a quantitatively reproducible framework, which is particularly important for cross‐study meta‐analysis. However, analyzing clinical MRI cohorts using automated tools can introduce bias if the segmentation technique does not accurately delineate regions in clinical cohorts; if the tools perform more poorly on clinical cohorts than control cohorts, statistical distinctions between 2 groups may be an artificial result of the technique. Quality assurance steps should be put in place to mitigate these risks. Here we applied a tool previously evaluated in clinical cohorts with severe pathology that utilizes a library of clinical data as priors during the segmentation process^29^ and performed visual QC on all images and segmentations (see Fig 1). Although we excluded a number of poor segmentations, we did not exclude data that had minor errors in segmentation. We therefore note the risks of using automated tools in studies such as this; although the quality of the segmentations was high overall, and we found no evidence for different performance of GIF between our groups (both clinical versus control and between different sites), we must remain cautious about the use of these tools in large cohort studies. The choice of segmentation tool will quantitatively influence the resulting segmentation, and thus future work should investigate the reproducibility of the current findings with other image processing tools.

In this analysis, we assume that PreHD and manifest HD participants are at the same disease stage between cohorts. However, because each study had different inclusion criteria, there are likely to be systematic differences between the cohorts. The demographics indicate that the cohorts have slightly differing characteristics, most notably in disease‐related factors such as UHDRS‐TMS, CAG repeat length, and DBS. Despite this, our analysis shows that regional imaging volumes follow a mostly consistent pattern of significant between‐group differences across all studies, after adjusting for covariates. Furthermore, although significant differences were detected for most measurements among cohorts, the regions with the largest group differences were consistent. Measurements from the single‐center IMAGE‐HD study showed fewer significant differences compared with the other studies, likely due to smaller sample size.

Finally, although imaging markers showed greater power than clinical variables, we note that this analysis was focused on PreHD individuals, who generally exhibit limited clinical symptoms. To date, clinical endpoints have been used as primary endpoints in HD trials due to their relative ease of administration and their functional relevance.^42^ However, clinical endpoints often include rater bias, as a participant's status and previous ratings are typically known beforehand. Composite behavioral measures tend to show less noise than individual measures, and the composite UHDRS has been used regularly in HD studies^43^; it was excluded from the current study as TFC data were not available for all 3 cohorts. In contrast to clinical measures, although sMRI measures reduce rater bias and are strongly predictive of clinical progression in HD,^25^ it is difficult to predict and detect the effect of treatment on global imaging measures.^42^ Fluid biomarkers in HD have recently shown a rapid treatment response and strong associations with clinical and neuroimaging markers.^44^ However, further validation of fluid biomarkers and their stability is required, and thus imaging markers are currently strong candidates for clinical trial endpoints.

This analysis is the first to directly compare 3 large HD imaging datasets and provide data‐driven sample size estimates using multistudy data. The results suggest that, for sMRI measures, there are key regions that show consistent effect sizes across multiple studies. We found that the region with the largest effect size for each study differed due to differences in inclusion criteria and thus mean cohort disease stage. Importantly, caudate volume had the highest power and lowest required number of participants required to reach this power. We therefore propose that caudate volume should be a key focus in future phase 1 and phase 2 trials. This analysis demonstrates that sMRI markers are generally robust to both participant and study differences and confirms their potential use as clinical trial endpoints in HD and more generally to other progressive neurodegenerative diseases.

## Author Contributions

P.A.W., E.B.J., A.E., S.G., and R.I.S. contributed to the conception and design of the study. P.A.W., E.B.J., A.E., S.G., H.J.J., G.R.P., A.M., C.S., N.G.‐K., J.S.P., S.J.T., R.I.S., and D.C.A. contributed to the acquisition and analysis of data; and P.A.W., E.B.J., L.A., and D.C.A. contributed to drafting the text and preparing the figures.

## Potential Conflicts of Interest

Nothing to report.

## Supporting information


**Appendix** **S1**: Supporting Information

## References

[1] Rodrigues FB , Wild EJ . Huntington's Disease Clinical Trials Corner: August 2018. J Huntingtons Dis 2018;7:279–286. 30103342 10.3233/JHD-189003 PMC6087448

[2] Georgiou‐Karistianis N , Scahill R , Tabrizi SJ , et al. Structural MRI in Huntington's disease and recommendations for its potential use in clinical trials. Neurosci Biobehav Rev 2013;37:480–490. 23376047 10.1016/j.neubiorev.2013.01.022

[3] Aylward EH , Li Q , Stine OC , et al. Longitudinal change in basal ganglia volume in patients with Huntington's disease. Neurology 1997;48:394–399. 9040728 10.1212/wnl.48.2.394

[4] Aylward EH , Sparks BF , Field KM , et al. Onset and rate of striatal atrophy in preclinical Huntington disease. Neurology 2004;63:66–72. 15249612 10.1212/01.wnl.0000132965.14653.d1

[5] Domínguez, JF , Egan GF , Gray MA , et al. Multi‐modal neuroimaging in premanifest and early Huntington's disease: 18 month longitudinal data from the IMAGE‐HD study. PLoS One 2013;8:e74131. 24066104 10.1371/journal.pone.0074131 PMC3774648

[6] Hobbs NZ , Farmer RE , Rees EM , et al. Short‐interval observational data to inform clinical trial design in Huntington's disease. J Neurol Neurosurg Psychiatry 2015;86:1291–1298. 25669748 10.1136/jnnp-2014-309768

[7] Majid DSA , Aron AR , Thompson W , et al. Basal ganglia atrophy in prodromal Huntington's disease is detectable over one year using automated segmentation. Move Disord 2011;26:2544–2551. 10.1002/mds.23912 PMC5615846 21932302

[8] Nopoulos PC , Aylward EH , Ross CA , et al. Cerebral cortex structure in prodromal Huntington disease. Neurobiol Dis 2010;40:544–554. 20688164 10.1016/j.nbd.2010.07.014 PMC2955824

[9] Paulsen JS , Nopoulos PC , Aylward, EH , et al. Striatal and white matter predictors of estimated diagnosis for Huntington disease. Brain Res Bull 2010;82:201–207. 20385209 10.1016/j.brainresbull.2010.04.003 PMC2892238

[10] Tabrizi SJ , Langbehn DR , Leavitt BR , et al. Biological and clinical manifestations of Huntington's disease in the longitudinal TRACK‐HD study: cross‐sectional analysis of baseline data. Lancet Neurol 2009;8:791–801. 19646924 10.1016/S1474-4422(09)70170-X PMC3725974

[11] van den Bogaard SJA , Dumas EM , Acharya TP , et al. Early atrophy of pallidum and accumbens nucleus in Huntington's disease. J Neurol 2010;258:412–420. 20936300 10.1007/s00415-010-5768-0 PMC3112014

[12] Tabrizi SJ , Reilmann R , Roos R , et al. Potential endpoints for clinical trials in premanifest and early Huntington's disease in the TRACK‐HD study: analysis of 24 month observational data. Lancet Neurol 2012;11:42–53. 22137354 10.1016/S1474-4422(11)70263-0

[13] Aylward EH , Anderson NB , Bylsma FW , et al. Frontal lobe volume in patients with Huntington's disease. Neurology 1998;50:252–258. 9443488 10.1212/wnl.50.1.252

[14] Douaud G , Gaura V , Ribeiro, M‐J , et al. Distribution of grey matter atrophy in Huntington's disease patients: a combined ROI‐based and voxel‐based morphometric study. Neuroimage 2006;32:1562–1575. 16875847 10.1016/j.neuroimage.2006.05.057

[15] Gómez‐Ansón B , Alegret M , Muñoz E , et al. Prefrontal cortex volume reduction on MRI in preclinical Huntington's disease relates to visuomotor performance and CAG number. Parkinsonism Relate Disord 2009;15:213–219. 10.1016/j.parkreldis.2008.05.010 18632301

[16] Sormani MP , Rovaris M , Valsasina P , et al. Measurement error of two different techniques for brain atrophy assessment in multiple sclerosis. Neurology 2004;62:1432–1434. 15111692 10.1212/01.wnl.0000120663.85143.b3

[17] Thieben MJ , Duggins AJ , Good CD , et al. The distribution of structural neuropathology in pre‐clinical Huntington's disease. Brain 2002;125:1815–1828. 12135972 10.1093/brain/awf179

[18] Wolf RC , Sambataro F , Vasic N , et al. Visual system integrity and cognition in early Huntington's disease. Eur J Neurosci 2014;40:2417–2426. 24698429 10.1111/ejn.12575

[19] Wijeratne PA , Young AL , Oxtoby NP , et al. An image‐based model of brain volume biomarker changes in Huntington's disease. Ann Clin Transl Neurol 2018;5:570–582. 29761120 10.1002/acn3.558 PMC5945962

[20] Paulsen JS , Langbehn DR , Stout JC , et al. Detection of Huntington's disease decades before diagnosis: the Predict‐HD study. J Neurol Neurosurg Psychiatry 2008;79:874–880. 18096682 10.1136/jnnp.2007.128728 PMC2569211

[21] Georgiou‐Karistianis N , Poudel GR , Domínguez D JF , et al. Functional and connectivity changes during working memory in Huntington's disease: 18 month longitudinal data from the IMAGE‐HD study. Brain Cogn 2013;83:80–91. 23938592 10.1016/j.bandc.2013.07.004

[22] Despotović I , Goossens B , Philips W . MRI segmentation of the human brain: challenges, methods, and applications. Comput Math Methods Med 2015;2015:450341. 25945121 10.1155/2015/450341 PMC4402572

[23] Huntington Study Group . Unified Huntington's Disease Rating Scale: reliability and consistency. Mov Disord 1996;11:136–142. 8684382 10.1002/mds.870110204

[24] Langbehn DR , Brinkman R , Falush D , et al. A new model for prediction of the age of onset and penetrance for Huntington's disease based on CAG length. Clin Genet 2004;65:267–277. 15025718 10.1111/j.1399-0004.2004.00241.x

[25] Tabrizi SJ , Scahill RI , Owen G , et al. Predictors of phenotypic progression and disease onset in premanifest Huntington's disease in the TRACK‐HD study: analysis of 36‐month observational data. Lancet Neurol 2013;12:637–649. 23664844 10.1016/S1474-4422(13)70088-7

[26] Smith A. Symbol Digit Modalities Test. Los Angeles, CA: Western Psychological Services, 1991.

[27] MacLeod CM . Half a century of research on the Stroop effect: an integrative review. Psychol Bull 1991;109:163–203. 2034749 10.1037/0033-2909.109.2.163

[28] Penney JB , Vonsattel JP , MacDonald ME , et al. CAG repeat number governs the development rate of pathology in Huntington's disease. Ann Neurol 1997;41:689–692. 9153534 10.1002/ana.410410521

[29] Cardoso MJ , Modat M , Wolz R , et al. Geodesic information flows: spatially‐variant graphs and their application to segmentation and fusion. IEEE Trans Med Imaging 2015;34:1976–1988. 25879909 10.1109/TMI.2015.2418298

[30] Eshaghi A , Prados F , Brownlee WJ , et al. Deep gray matter volume loss drives disability worsening in multiple sclerosis. Ann Neurol 2018;83:210–222. 29331092 10.1002/ana.25145 PMC5838522

[31] Gelman A , Hill J . Data analysis using regression and multilevel/hierarchical models. Cambridge, United Kingdom: Cambridge University Press, 2006.

[32] Raudenbush SW , Liu X . Statistical power and optimal design for multisite randomized trials. Psychol Methods 2000;5:199–213. 10937329 10.1037/1082-989x.5.2.199

[33] *R Core Team. R: A language and environment for statistical computing. Available at*: http://www.r-project.org/. Accessed March 13, 2020.

[34] Bates D , Maechler M , Bolker B , Walker S . Fitting linear mixed‐effects models using lme4. J Stat Softw 2015;67:1–48.

[35] Cohen J. Statistical power analysis for the behavioral sciences. 2nd ed. Hillsdale, NJ: Erlbaum, 1988.

[36] Jones SR , Carley S , Harrison M . An introduction to power and sample size estimation. Emerg Med J 2003;20:453–458. 12954688 10.1136/emj.20.5.453 PMC1726174

[37] Paulsen JS , Long JD , Johnson HJ , et al. Clinical and biomarker changes in premanifest Huntington disease show trial feasibility: a decade of the PREDICT‐HD study. Front Aging Neurosci 2014;6:78. 24795630 10.3389/fnagi.2014.00078 PMC4000999

[38] Ahveninen LM , Stout JC , Georgiou‐Karistianis N , Lorenzetti V , Glikmann‐Johnston Y . Reduced amygdala volumes are related to motor and cognitive signs in Huntington's disease: The IMAGE‐HD study. Neuroimage Clin 2018;18:881–887. 10.1016/j.nicl.2018.03.027. 29876272 PMC5988225

[39] Bogaard SJA , Dumas EM , Acharya TP , et al. Early atrophy of pallidum and accumbens nucleus in Huntington's disease. J Neurol 2011;258:412–420. 20936300 10.1007/s00415-010-5768-0 PMC3112014

[40] Singh‐Bains MK , Waldvogel, HJ , Faull RLM . The role of the human globus pallidus in Huntington's disease. Brain Pathol 2016;26:741–751. 27529459 10.1111/bpa.12429 PMC8029019

[41] Oxtoby NP , Alexander DC . Imaging plus X: multimodal models of neurodegenerative disease. Curr Opin Neurol 2017;30:371–379. 28520598 10.1097/WCO.0000000000000460 PMC5491241

[42] Weir DW , Sturrock A , Leavitt BR . Development of biomarkers for Huntington's disease. Lancet Neurol 2011;10:573–590. 21601164 10.1016/S1474-4422(11)70070-9

[43] Schobel SA , Palermo G , Auinger P , et al. Motor, cognitive, and functional declines contribute to a single progressive factor in early HD. Neurology 2017;89:2495–2502. 29142089 10.1212/WNL.0000000000004743 PMC5729794

[44] Byrne LM , Rodrigues FB , Blennow K , et al. Neurofilament light protein in blood as a potential biomarker of neurodegeneration in Huntington's disease: a retrospective cohort analysis. Lancet Neurol 2017;16:601–609. 28601473 10.1016/S1474-4422(17)30124-2 PMC5507767

