# Supplementary material for: Robust Markers and Sample Sizes for Multicenter Trials of Huntington Disease
Source: Ann Neurol. 2020 Mar 14;87(5):751–62. doi: 10.1002/ana.25709 (PMC7187160; doi:10.1002/ana.25709)
Supplement: Supplementary file 1 — Appendix S1: Supporting Information [file ANA-87-751-s001.docx]

**Robust markers and sample sizes for multi-centre trials of Huntington’s disease**

**Supplementary Material**

Peter A. Wijeratne, PhD**^1^***, Eileanoir B. Johnson, PhD**^2^***, Arman Eshaghi, MD, PhD**^1,3^**, Leon Aksman, PhD**^1^**, Sarah Gregory, PhD**^2^**, Hans J. Johnson, PhD**^4,5^**, Govinda R. Poudel, PhD**^6^**, Amrita Mohan, PhD**^7^**, Cristina Sampaio, MD, PhD**^7^**, Nellie Georgiou-Karistianis, PhD**^8^**, Jane S. Paulsen, PhD**^4^**, Sarah J. Tabrizi, MD, PhD**^2^**, Rachael I. Scahill, PhD**^2^**, the IMAGE-HD, PREDICT-HD, and TRACK-HD investigators, Daniel C. Alexander, PhD**^1^**

Supplementary Table 1: PREDICT-HD Scanner Acquisition Parameters

| Site | Field Strength (Tesla) | TR (ms) | TE (ms) | Flip Angle (°) | Voxel Resolution (mm x mm x mm) | Manufacturer | Model |
| --- | --- | --- | --- | --- | --- | --- | --- |
| 11 | 1.5 | 18 | 3 | 20 | 0.9375x0.9375x1.5 | GE | GENESIS_SIGNA |
| 11 | 1.5 | 18 | 3 | 20 | 0.9375x0.9375x1.5 | GE | SIGNA |
| 11 | 1.5 | 18 | 3 | 20 | 0.9375x0.9375x1.5 | GE | SIGNAEXCITE |
| 11 | 1.5 | 500 | 11 | 90 | 0.9375x0.9375x3 | GE | SIGNAHDx |
| 11 | 1.5 | 18 | 3 | 20 | 0.9375x0.9375x1.5 | GE | SignaHDxt |
| 12 | 1.5 | 18 | 3 | 20 | 0.9375x0.9375x1.5 | GE | GENESIS_SIGNA |
| 12 | 3 | 2300 | 2.87 | 10 | 1.1015625x1.1015625x1.1 | Siemens | TrioTim |
| 12 | 1.5 | 18 | 4.75 | 20 | 0.9375x0.9375x1.5 | Siemens | Avanto |
| 15 | 1.5 | 18 | 4.17 | 20 | 0.9375x0.9375x1.5 | Siemens | Sonata |
| 18 | 3 | 7.69 | 3.5 | 8 | 1.0714285x1.0714285x1.1 | Philips | Achieva |
| 18 | 1.5 | 18 | 3 | 20 | 0.9375x0.9375x1.5 | GE | GENESIS_SIGNA |
| 18 | 1.5 | 18 | 3 | 20 | 0.9375x0.9375x1.5 | GE | SIGNAEXCITE |
| 22 | 1.5 | 18 | 3 | 20 | 0.9375x0.9375x1.5 | GE | SignaHDxt |
| 22 | 1.5 | 18 | 3 | 20 | 0.9375x0.9375x1.5 | GE | SIGNAHDx |
| 22 | 1.5 | 18 | 4 | 20 | 0.9375x0.9375x1.5 | GE | GENESIS_SIGNA |
| 22 | 1.5 | 18 | 3 | 20 | 0.9375x0.9375x1.5 | GE | SIGNAEXCITE |
| 24 | 1.5 | 18 | 3 | 20 | 0.9375x0.9375x1.5 | GE | SIGNA |
| 24 | 1.5 | 18 | 3 | 20 | 0.9375x0.9375x1.5 | GE | SIGNAEXCITE |
| 24 | 3 | 7.78 | 2.976 | 12 | 1x1x1 | GE | SIGNAHDx |
| 24 | 3 | 7.772 | 2.976 | 12 | 1x1x1 | GE | SignaHDxt |
| 40 | 1.5 | 18 | 3 | 20 | 0.9375x0.9375x1.5 | GE | GENESIS_SIGNA |
| 40 | 1.5 | 18 | 3 | 20 | 0.9375x0.9375x1.5 | GE | SIGNA |
| 40 | 3 | 2300 | 2.87 | 10 | 1.1015625x1.1015625x1.1 | Siemens | TrioTim |
| 42 | 1.5 | 18 | 3 | 20 | 0.9375x0.9375x1.5 | GE | GENESIS_SIGNA |
| 42 | 1.5 | 18 | 3 | 20 | 0.937489x0.937489x1.5 | GE | SIGNAEXCITE |
| 42 | 3 | 2300 | 1.91 | 10 | 1.1015625x1.1015625x1.1 | Siemens | TrioTim |
| 43 | 3 | 6.652 | 2.82 | 12 | 1x1x1 | GE | SignaHDxt |
| 43 | 1.5 | 18 | 3 | 20 | 0.9375x0.9375x1.5 | GE | SIGNAHDx |
| 43 | 1.5 | 18 | 3 | 20 | 0.9375x0.9375x1.5 | GE | SIGNAEXCITE |
| 43 | 1.5 | 17.3 | 7.788 | 20 | 0.468755x0.46875x1.5 | GE | GENESIS_SIGNA |
| 43 | 1.5 | 18 | 3.01 | 20 | 0.9375x0.9375x1.5 | Siemens | Avanto |
| 45 | 1.5 | 18 | 3 | 20 | 0.9375x0.9375x1.5 | GE | GENESIS_SIGNA |
| 45 | 3 | 2300 | 2.87 | 10 | 1.1015625x1.1015625x1.1 | Siemens | TrioTim |
| 48 | 1.5 | 3.7688 | 1.6997 | 8 | 0.9765625x0.9765625x2.2 | Philips | Achieva |
| 48 | 1.5 | 18 | 4.75 | 20 | 0.9375x0.9375x1.5 | Siemens | Avanto |
| 48 | 1.5 | 18 | 3 | 20 | 0.9375x0.9375x1.5 | GE | GENESIS_SIGNA |
| 48 | 1.5 | 18 | 3 | 20 | 0.9375x0.9375x1.5 | GE | SIGNA |
| 51 | 1.5 | 18 | 3 | 20 | 0.9375x0.9375x1.5 | GE | GENESIS_SIGNA |
| 51 | 1.5 | 18 | 3 | 20 | 0.9375x0.9375x1.5 | GE | SIGNAEXCITE |
| 58 | 1.5 | 18 | 4.38 | 20 | 0.9375x0.9375x1.5 | Siemens | Symphony |
| 58 | 1.5 | 18 | 3 | 20 | 0.9375x0.9375x1.5 | GE | SIGNAEXCITE |
| 58 | 1.5 | 18 | 4.38 | 20 | 0.9375x0.9375x1.5 | Siemens | Avanto |
| 58 | 3 | 6.437 | 2.919 | 8 | 1.0666667x1.0666667x1.1 | Philips | Achieva |
| 58 | 1.5 | 18 | 3 | 20 | 0.9375x0.9375x1.5 | GE | SIGNA |
| 68 | 1.5 | 9.3 | 1.976 | 20 | 0.937507x0.937494x1.5 | GE | GENESIS_SIGNA |
| 68 | 1.5 | 1880 | 3.67 | 20 | 0.9375x0.9375x1.5 | Siemens | Sonata |
| 70 | 1.5 | 18 | 3 | 20 | 0.9375x0.9375x1.5 | GE | GENESIS_SIGNA |
| 70 | 1.5 | 18 | 3 | 20 | 0.937506x0.937512x1.5 | GE | SIGNAEXCITE |
| 86 | 1.5 | 18 | 3 | 20 | 0.9375x0.9375x1.5 | GE | GENESIS_SIGNA |
| 87 | 1.5 | 18 | 3 | 20 | 0.9375x0.9375x1.5 | GE | GENESIS_SIGNA |
| 87 | 3 | 2300 | 2.87 | 10 | 1.1015625x1.1015625x1.1 | Siemens | TrioTim |
| 91 | 1.5 | 18 | 4.4 | 20 | 0.9375x0.9375x1.5 | Siemens | Symphony |
| 91 | 1.5 | 18 | 4.4 | 20 | 0.9375x0.9375x1.5 | Siemens | SymphonyTim |
| 92 | 1.5 | 18 | 3 | 20 | 0.9375x0.9375x1.5 | Siemens | Sonata |
| 92 | 3 | 2300 | 2.87 | 10 | 1.1015625x1.1015625x1.1 | Siemens | TrioTim |
| 93 | 1.5 | 18 | 3 | 20 | 0.9375x0.9375x1.5 | GE | SIGNAEXCITE |
| 93 | 1.5 | 18 | 3 | 20 | 0.9375x0.9375x1.5 | GE | GENESIS_SIGNA |

Acquisition parameters for the 13 PREDICT-HD scanners included in the current study. Total number of scans from each scanner: Achieva = 6, Avanto = 31, Espree = 1, GENESIS_SIGNA = 312, SIGNA = 69, SIGNAEXCITE = 130, SIGNAHDx = 39, SignaHDxt = 3, Sonata = 43, Symphony = 10, SymphonyTim = 1, TrioTim = 138, Verio = 12.

Supplementary Table 2: Analysis Cut Flow

|  | Total at baseline | Data present at baseline + two follow-ups | Passed through segmentation pipeline without error | Same scanner and field strength at all time points | Passed visual quality control |
| --- | --- | --- | --- | --- | --- |
| PREDICT-HD | 1000 | 482 | 482 | 301 | 265 |
| TRACK-HD | 358 | 295 | 294 | 294 | 294 |
| IMAGE-HD | 96 | 67 | 67 | 67 | 65 |

Number of participants in each cohort at each stage of selection.

Supplementary Table 3: Follow-up One Demographics of Participants

|  | Control participants | | | PreHD participants | | | | HD participants | | | |
| --- | --- | --- | --- | --- | --- | --- | --- | --- | --- | --- | --- |
|  | PREDICT-HD | TRACK-HD | IMAGE-HD | | PREDICT-HD | TRACK-HD | IMAGE-HD | | PREDICT-HD | TRACK-HD | IMAGE-HD |
| N | 56 | 106 | 23 | | 205 | 105 | 22 | | 4 | 83 | 20 |
| Age | 45.1  ±12.1 | 46.3  ±10.2 | 44.4  ±13.9 | | 41.6  ±11 | 41.1  ±8.8 | 43.4  ±8.3 | | 44.3  ±7.9 | 49.1  ±9.5 | 53.4  ±8.8 |
| Sex | 36:20 | 61:45 | 16:7 | | 116:70 | 56:49 | 16:6 | | 16:7 | 44:39 | 7:13 |
| TIV | 1.37  ±0.13 | 1.39  ±0.13 | 1.44  ±0.14 | | 1.37  ±0.13 | 1.4  ±0.146 | 1.34  ±0.14 | | 1.34  ±0.12 | 1.37  ±0.12 | 1.41  ±0.15 |
| CAG | 20.3  ±3.3 | - | - | | 42.1  ±2.5 | 43  ±2.3 | 42  ±2 | | 43.5  ±3 | 43.6  ±3.1 | 42.9  ±2.4 |
| TMS | 2.2  ±2.6 | 1.3  ±1.6 | - | | 5.6  ±5.4 | 4.6  ±3.3 | 2.8  ±3.7 | | 20.2  ±8.7 | 27.6  ±13.5 | 21.8  ±11.3 |
| DCL | 0.4  ±0.6 | 0.3  ±0.4 | - | | 1  ±0.8 | 0.6  ±0.6 | - | | 3.8  ±0.7 | 4  ±0.1 | - |
| TFC | 13  ±0 | 12.3  ±1.4 | - | | 12.6  ±1.7 | 12.2  ±1.6 | - | | 11.7  ±1.9 | 12.2  ±1.6 | - |
| DBS | - | - | - | | 267.1  ±69 | 300.3  ±49 | 282.3  ±58 | | 343.6  ±82 | 382.5  ±80 | 388.5  ±78 |

Demographic data for the PREDICT-HD, TRACK-HD and IMAGE-HD participants at follow-up one. A value of ‘-’ indicates that the data were not available. Acronyms used: TIV = total intracranial volume (units: litres); CAG = cytosine, adenine, guanine; TMS = total motor score; DCL = diagnostic confidence limit; TFC = total functional capacity; DBS = disease burden score (units: CAG x years).

Supplementary Table 4: Follow-up Two Demographics of Participants

|  | Control participants | | | PreHD participants | | | | HD participants | | | |
| --- | --- | --- | --- | --- | --- | --- | --- | --- | --- | --- | --- |
|  | PREDICT-HD | TRACK-HD | IMAGE-HD | | PREDICT-HD | TRACK-HD | IMAGE-HD | | PREDICT-HD | TRACK-HD | IMAGE-HD |
| N | 56 | 106 | 23 | | 205 | 105 | 22 | | 4 | 83 | 20 |
| Age | 45.1  ±12.1 | 46.3  ±10.2 | 44.4  ±13.9 | | 41  ±10.9 | 41.1  ±8.8 | 43.4  ±8.3 | | 45.7  ±9.4 | 49.1  ±9.5 | 53.4  ±8.8 |
| Sex | 36:20 | 61:45 | 16:7 | | 103:67 | 56:49 | 16:6 | | 29:10 | 44:39 | 7:13 |
| TIV | 1.37  ±0.13 | 1.39  ±0.13 | 1.44  ±0.14 | | 1.37  ±0.13 | 1.4  ±0.15 | 1.34  ±0.14 | | 1.34  ±0.12 | 1.37  ±0.12 | 1.41  ±0.16 |
| CAG | 20.3  ±3.3 | - | - | | 42.1  ±2.5 | 43  ±2.3 | 42  ±2 | | 43.1  ±2.9 | 43.6  ±3.1 | 42.9  ±2.4 |
| TMS | 2.5  ±3.2 | 1.4  ±1.8 | - | | 5.7  ±5.5 | 5.6  ±4.4 | 3.2  ±4.7 | | 21.5  ±9.8 | 29  ±13 | 21.9  ±9.9 |
| DCL | 0.4  ±0.7 | 0.3  ±0.4 | - | | 1.1  ±0.9 | 0.6  ±0.6 | - | | 3.7  ±0.8 | 4  ±0.1 | - |
| TFC | 12.4  ±2.6 | 12.4  ±1.3 | - | | 12.4  ±2.3 | 12.4  ±1.4 | - | | 11  ±2.7 | 12.2  ±1.7 | - |
| DBS | - | - | - | | 268.6  ±68 | 307.9  ±51 | 289.1  ±60 | | 343.5  ±84 | 390.9  ±83 | 395.9  ±80 |

Demographic data for the PREDICT-HD, TRACK-HD and IMAGE-HD participants at follow-up two. A value of ‘-’ indicates that the data were not available. Acronyms used: TIV = total intracranial volume (units: litres); CAG = cytosine, adenine, guanine; TMS = total motor score; DCL = diagnostic confidence limit; TFC = total functional capacity; DBS = disease burden score (units: CAG x years).

Supplementary Table 5: Inter-Study P-values

|  | HC_PvT | HC_PvI | HC_TvI | PRE_PvT | PRE_PvI | PRE_TvI | HD_PvT | HD_PvI | HD_TvI |
| --- | --- | --- | --- | --- | --- | --- | --- | --- | --- |
| Age | 0.53 | 0.83 | 0.54 | 0.54 | 0.43 | 0.27 | 0.7 | 0.31 | 0.06 |
| Sex | 0.41 | 0.66 | 0.3 | 0.11 | 0.35 | 0.08 | 0.45 | 0.21 | 0.15 |
| TIV | 0.34 | 0.06 | 0.17 | 0.04 | 0.38 | 0.07 | 0.55 | 0.29 | 0.27 |
| CAG | - | - | - | 0.001 | 0.62 | 0.05 | 0.8 | 0.95 | 0.26 |
| TMS | 0.001 | <0.001 | <0.001 | <0.001 | <0.001 | <0.001 | 0.78 | 0.68 | 0.05 |
| DCL | 0.07 | - | - | 0.002 | - | - | 0.32 | - | - |
| TFC | <0.001 | - | - | 0.04 | - | - | 0.78 | - | - |
| DBS | - | - | - | <0.001 | 0.7 | 0.11 | 0.42 | 0.4 | 0.9 |

P-values from inter-study t-tests in demographic data at baseline. Acronyms used: HC = healthy control, PRE = preHD, HD = manifest HD, PvT = PREDICT vs TRACK, PvI = PREDICT vs IMAGE, TvI = TRACK vs IMAGE; TIV = total intracranial volume; CAG = cytosine, adenine, guanine; TMS = total motor score; DCL = diagnostic confidence limit; TFC = total functional capacity; DBS = disease burden score.

Supplementary Table 6: Raw Regional Volumes

| Biomarker | HC_P | HC_T | HC_I | PRE_P | PRE_T | PRE_I | HD_P | HD_T | HD_I |
| --- | --- | --- | --- | --- | --- | --- | --- | --- | --- |
| Non-ventricular CSF | 237005.47  ±50966.07 | 242479.29  ±50348.86 | 253193.37  ±54444.08 | 244643.34  ±49646.06 | 259384.69  ±55605.29 | 232047.16  ±47315.8 | 256552.01  ±57853.28 | 269975.12  ±52726.55 | 282085.52  ±67146.08 |
| 3rd Ventricle | 1136.07  ±479.43 | 1182.81  ±573.86 | 1215.25  ±558.24 | 1248.04  ±485.77 | 1359.54  ±576.75 | 1167.86  ±418.95 | 1445.95  ±658.67 | 1873.75  ±733.14 | 2121.98  ±901.81 |
| 4th Ventricle | 2078.27  ±689.39 | 1896.41  ±577.94 | 1969.18  ±536.1 | 2034.3  ±631.55 | 1982.68  ±658.88 | 2117.06  ±570.5 | 1930.22  ±577.52 | 2016.62  ±635.35 | 2197.17  ±719.52 |
| 5th Ventricle | 2.89  ±2.79 | 3.17  ±2.36 | 4.21  ±2.95 | 2.9  ±2.39 | 3.13  ±3.07 | 2.84  ±2.05 | 2.5  ±2.5 | 2  ±1.52 | 1.65  ±1.98 |
| Accumbens Area | 605.42  ±128.9 | 641.1  ±131.56 | 700.53  ±154.72 | 593.12  ±134.09 | 618.44  ±142.53 | 568.8  ±140.29 | 552.47  ±120.95 | 578.09  ±115.45 | 651.05  ±174.62 |
| Amygdala | 2656.66  ±535.82 | 2894.61  ±561.2 | 2944.4  ±526.6 | 2608.75  ±524.62 | 2819.17  ±596.49 | 2455.92  ±521.11 | 2407.38  ±473.57 | 2518.52  ±441.69 | 2550.61  ±524.32 |
| Pons | 9421.53  ±2404.68 | 9494.23  ±2056.6 | 10468.55  ±2216.55 | 8961.22  ±1905.99 | 9195.05  ±2165.73 | 8845.9  ±2117.76 | 7973.16  ±1391.21 | 8283.12  ±2028.83 | 9911.65  ±2567.25 |
| Brain Stem | 8203.57  ±1582.72 | 8551.92  ±1542.13 | 8855.9  ±1745.15 | 8077.19  ±1571.01 | 8529.22  ±1771.19 | 7746.64  ±1581.17 | 7375.88  ±1133.68 | 7883.95  ±1428.52 | 8524.96  ±2050.36 |
| Caudate | 3609.91  ±786.72 | 3712.32  ±802.73 | 3832  ±828.45 | 3268.59  ±738.98 | 3349.12  ±801.61 | 3133.2  ±805.03 | 2775.11  ±651.51 | 2787.01  ±597.57 | 3237.7  ±942.84 |
| Cerebellum Exterior | 90718.97  ±16826.76 | 92803.22  ±16246.31 | 94724.17  ±17017.7 | 93822.78  ±17194.43 | 94484.37  ±18914.58 | 87440.54  ±15979.23 | 88502.55  ±14680.15 | 90720.11  ±15427.79 | 96484.83  ±18378.17 |
| Cerebellum White Matter | 33440.21  ±9369.7 | 33495.54  ±6422.46 | 41846.4  ±9079.71 | 32258.06  ±7890.39 | 33646.9  ±7018.22 | 35678.63  ±8034.8 | 29702.05  ±6732.31 | 33190.88  ±6242.89 | 43136.05  ±11381.6 |
| Cerebral Exterior | 9.02  ±6 | 11.28  ±6.07 | 12.49  ±4.63 | 10.78  ±5.74 | 12.89  ±6.93 | 11.56  ±5.33 | 11.65  ±6.75 | 17.33  ±8.5 | 21.13  ±11.92 |
| 3rd Ventricle (Posterior part) | 1263.1  ±323.14 | 1348.2  ±353.04 | 1246.9  ±394.7 | 1365.21  ±312.78 | 1475.87  ±420.53 | 1150  ±270 | 1436.7  ±394.59 | 1616.27  ±431.6 | 1688.08  ±436.82 |
| Hippocampus | 6096.1  ±1112.79 | 6567.56  ±1181.1 | 6555.62  ±1125.67 | 6028.97  ±1074.66 | 6529.65  ±1226.89 | 5829.92  ±1148.8 | 5546.98  ±897.29 | 6029.28  ±931.61 | 5916.53  ±1190.56 |
| Inf Lat Vent | 712.77  ±260.07 | 750.11  ±337.12 | 946.57  ±314.04 | 808.59  ±353.02 | 809.93  ±341.43 | 771.25  ±248.78 | 743.4  ±458.18 | 1067.14  ±696.17 | 1136.94  ±545.92 |
| Lateral Ventricle | 19569.06  ±11272.49 | 21478.38  ±11751.26 | 21916.83  ±10896.48 | 21270.45  ±10851.62 | 22705.66  ±10186.28 | 19864.81  ±9585.25 | 24775.41  ±13409.15 | 33782.22  ±18309.44 | 38267.4  ±23199.85 |
| Pallidum | 3322.22  ±660.3 | 3273.78  ±641.22 | 3840.99  ±855.02 | 3077.54  ±648.91 | 2984.54  ±654.62 | 3113.52  ±737.5 | 2580.97  ±449.33 | 2539.73  ±490.07 | 3147.72  ±755.97 |
| Putamen | 5892.83  ±1066.7 | 5543.54  ±1180.82 | 6429.51  ±1452.92 | 5609.02  ±1154.08 | 5186.27  ±1120.93 | 5300  ±1363.09 | 4988.81  ±906.55 | 4787.59  ±899.6 | 5672.32  ±1444.86 |
| Thalamus Proper | 7791.26  ±1475.81 | 8241.87  ±1686.32 | 7984.99  ±1413.53 | 7816.28  ±1723.84 | 8228.12  ±1776.9 | 7054.58  ±1446.26 | 7358.27  ±1613.83 | 7716.76  ±1430.94 | 7775.14  ±1759.82 |
| Ventral DC | 5758.29  ±1070.05 | 5769.52  ±1124.69 | 6381.5  ±1336.79 | 5701.82  ±1109.42 | 5757.75  ±1256.63 | 5727.88  ±1399 | 5210.59  ±886.89 | 5355.72  ±1055.55 | 6206.65  ±1661.16 |
| Ventricular Lining | 4942.89  ±1356.62 | 5454.06  ±1435.77 | 5519.61  ±1456.33 | 5023.59  ±1235.74 | 5513.8  ±1393.58 | 4720.71  ±1017.54 | 5363.55  ±1762.4 | 6733.49  ±2403.44 | 7009.94  ±2664.02 |
| Optic Chiasm | 84.5  ±23.59 | 98.79  ±25.47 | 87.51  ±23.59 | 89.14  ±22.77 | 106.57  ±25.06 | 87.04  ±17.22 | 102.17  ±28.07 | 126.3  ±36.19 | 129.4  ±40.02 |
| Cerebellar Vermal Lobules I-V | 3516.24  ±729.58 | 3642.33  ±748.57 | 3820.75  ±943.79 | 3726.16  ±731.63 | 3849.4  ±847.55 | 3231.99  ±697.84 | 3533.1  ±582.99 | 3732.18  ±723.56 | 3684.87  ±830.94 |
| Cerebellar Vermal Lobules VI-VII | 1523.5  ±269.37 | 1552.3  ±265.27 | 1558.68  ±303.16 | 1544.58  ±270.47 | 1566.59  ±306.57 | 1441.63  ±249.54 | 1475.54  ±239.02 | 1547.16  ±271.5 | 1637.49  ±298.45 |
| Cerebellar Vermal Lobules VIII-X | 2539.75  ±494.93 | 2567.22  ±467.3 | 2712.26  ±518.54 | 2607.7  ±502.44 | 2574.97  ±527.94 | 2479.02  ±492.76 | 2493.5  ±413.06 | 2560.35  ±452.9 | 2803.44  ±707.71 |
| Basal Forebrain | 1034.28  ±207.75 | 1118.8  ±209.16 | 1026.33  ±187.25 | 1004.6  ±195.04 | 1102.07  ±234.24 | 891.9  ±184.02 | 920.59  ±193.62 | 984.1  ±169.16 | 927.08  ±182.06 |
| Temporal White Matter | 73153.69  ±15991.74 | 74051.63  ±15701.55 | 83549.8  ±17942.66 | 71774.73  ±15878.07 | 74131.15  ±17913.74 | 70358.17  ±17566.17 | 66043.59  ±13652.08 | 68412.26  ±13225.54 | 76827.88  ±18943.59 |
| Insula White Matter | 29492.56  ±6210.82 | 28174.95  ±5646.96 | 33874.82  ±7292.28 | 28015.24  ±5726.33 | 27015.52  ±6008.52 | 28424.69  ±6573.4 | 24502.85  ±4091.45 | 24258.76  ±4703.37 | 29570.09  ±7214.68 |
| Cingulate White Matter | 7404.06  ±1884.43 | 7892.23  ±1836.24 | 9100.36  ±2061.9 | 7263.53  ±1687.79 | 7717.97  ±2021.55 | 7369.17  ±1947.47 | 6693.76  ±1743.68 | 6944.55  ±1564.84 | 7800.44  ±1991.26 |
| Frontal White Matter | 156643.71  ±36347.12 | 152839.49  ±33148.31 | 158707.64  ±32388.92 | 157406.87  ±35831.15 | 154244.46  ±36271.54 | 136046.33  ±33089.28 | 146823.94  ±29922.06 | 140162.98  ±27130.14 | 141125.64  ±34551.44 |
| Occipital White Matter | 50738.6  ±10501.09 | 50426.17  ±10646.6 | 54332.56  ±12651.19 | 50351.28  ±11658.57 | 49777.19  ±12036.92 | 45665.98  ±11709.55 | 46509.97  ±11455.34 | 46559.37  ±9231.35 | 50969.79  ±13683.09 |
| Parietal White Matter | 102093.38  ±22169.24 | 101898.6  ±20951.95 | 109315.85  ±23107.99 | 102352.74  ±22161.09 | 102508.2  ±23781.68 | 92800.33  ±23990.25 | 95817.24  ±19309.7 | 93461.86  ±18160.29 | 97360.4  ±23932.81 |
| Corpus Callosum | 18556.48  ±4570.33 | 19579.54  ±4482.69 | 21382.15  ±5379.12 | 18278.97  ±4064.62 | 19484.51  ±4695.36 | 17714.93  ±4378.09 | 16644.09  ±3330.88 | 17649.08  ±3696.65 | 19222  ±4731.82 |
| Claustrum | 5255.62  ±1001.75 | 5584.65  ±1154.2 | 5644.72  ±1235.73 | 5332.6  ±1101.04 | 5632.85  ±1146.88 | 4772.26  ±1108.98 | 4973.94  ±1001.21 | 5253.67  ±996.42 | 5115.87  ±1102.59 |
| Anterior cingulate gyrus | 9972.99  ±2105.87 | 9930.46  ±2216.83 | 10081.07  ±1974.56 | 9940.92  ±2128.41 | 10186.11  ±2529.19 | 8753.91  ±1842.79 | 9315  ±2044.07 | 9526.87  ±2024.54 | 9453.93  ±2091.11 |
| Anterior insula | 6959.86  ±1336.56 | 7448.77  ±1533.64 | 7438.34  ±1614.69 | 6978.07  ±1493.68 | 7491.99  ±1541.62 | 6320.89  ±1432.19 | 6293.23  ±1316 | 6520.46  ±1331.85 | 6399.47  ±1407.19 |
| Anterior orbital gyrus | 3102.98  ±655.52 | 3351.21  ±674.81 | 3350.97  ±633.46 | 3159.18  ±682.93 | 3460.62  ±754.43 | 2973.14  ±617.3 | 3013  ±489 | 3220.98  ±605.36 | 3290.44  ±665.55 |
| Angular gyrus | 15386.92  ±3225.32 | 15705.36  ±3124.94 | 16889.32  ±3462 | 15286.05  ±3341.14 | 15795.58  ±3495.37 | 14560.44  ±3324.71 | 13589.84  ±2643.73 | 14188.61  ±3110.34 | 15127.75  ±3641.6 |
| Calcarine cortex | 6554.3  ±1555.52 | 7197.61  ±1575.67 | 7301.98  ±1722.22 | 6325.07  ±1493.89 | 7127.53  ±1797.58 | 6393.14  ±1471.76 | 5340.4  ±1595.83 | 5952.26  ±1627.78 | 6107.65  ±1596.96 |
| Central operculum | 7812.39  ±1656.31 | 8154.63  ±1695.06 | 8408.67  ±1870.76 | 7867.68  ±1746.29 | 8512.95  ±1846.69 | 6991.75  ±1756.87 | 7243.26  ±1214.13 | 7602.73  ±1606.66 | 7648.72  ±1635.91 |
| Cuneus | 8499.29  ±1761.86 | 9305.93  ±2050.29 | 9750.48  ±1877 | 8184.87  ±2007.26 | 9243.64  ±2208.62 | 8203.92  ±1884.62 | 7033.28  ±1814.81 | 7983.92  ±1867.48 | 8189.92  ±2091.21 |
| Entorhinal area | 4426.19  ±889.77 | 4790.74  ±1008.02 | 4768.64  ±897.44 | 4363.02  ±928.9 | 4822.91  ±1061.09 | 4147.75  ±896.57 | 4200.85  ±856.81 | 4518.86  ±862.95 | 4548.37  ±996.52 |
| Frontal operculum | 3304.75  ±721.76 | 3553.99  ±803.94 | 3526.31  ±672.52 | 3323.85  ±775.55 | 3654.87  ±855.03 | 3056.29  ±769.77 | 2976.67  ±631.97 | 3220.8  ±620.8 | 3121.22  ±743.24 |
| Frontal pole | 4907.54  ±1719.19 | 4512.84  ±1054.13 | 6232.3  ±1487.78 | 4526.83  ±1393.15 | 4723.97  ±1248.08 | 5656.4  ±1303.87 | 3846.34  ±1041.38 | 4218.08  ±986.57 | 6192.65  ±1621.22 |
| Fusiform gyrus | 17159.4  ±3271.1 | 18012.39  ±3681.3 | 18372.76  ±3458.38 | 17246.76  ±3511.32 | 18390.75  ±4009.69 | 16028.51  ±3312.15 | 16200.29  ±3164.43 | 17310.7  ±3177.77 | 17239.17  ±3435.01 |
| Gyrus rectus | 3956.33  ±831.18 | 3680.54  ±800.77 | 3810.23  ±683 | 3962.35  ±801.43 | 3767.87  ±848.89 | 3361.57  ±648.8 | 3768.62  ±710.07 | 3563.05  ±694.35 | 3769.14  ±847.54 |
| Inferior occipital gyrus | 12405.47  ±2386.61 | 13547.75  ±2753.37 | 13786.47  ±2898.86 | 12221.9  ±2586.2 | 13695.93  ±3057.12 | 11537.36  ±2512.09 | 10937.27  ±2100.25 | 12034.45  ±2455.7 | 11973.72  ±2555.55 |
| Inferior temporal gyrus | 23972.33  ±4735.01 | 24319.01  ±4961.44 | 25053.14  ±4678.04 | 24040.8  ±4993.61 | 25000.65  ±5628.39 | 21419.08  ±4230.7 | 22706.46  ±4431.89 | 23238.5  ±4299.74 | 23553.8  ±5043.61 |
| Lingual gyrus | 14955.34  ±2844.43 | 16510.53  ±3209.62 | 16519.56  ±3031.3 | 14921.14  ±3040.35 | 16657.52  ±3690.18 | 14625.27  ±2782.54 | 13593.65  ±2848.84 | 15082.97  ±3020.07 | 14791.38  ±3275.45 |
| Lateral orbital gyrus | 3397.68  ±696.89 | 3514.1  ±675.76 | 3740.31  ±737.41 | 3463.09  ±739.14 | 3679  ±837.4 | 3397.27  ±712.09 | 3195.28  ±609.72 | 3331.1  ±604.69 | 3691.28  ±777.1 |
| Middle cingulate gyrus | 9308.4  ±1866.41 | 9817.08  ±1962.53 | 10171.94  ±1832.13 | 9594.17  ±1943.47 | 9920.98  ±2136.34 | 8447.58  ±1813.17 | 9167.89  ±1872.56 | 9539.3  ±1794.74 | 9243.43  ±1861.55 |
| Medial frontal cortex | 4652.9  ±918.55 | 4391.1  ±933.88 | 4454.21  ±947.99 | 4632.15  ±917.4 | 4473.13  ±1055.6 | 3927.99  ±829.32 | 4283.26  ±830.83 | 4126.7  ±839.56 | 4357.8  ±1070.49 |
| Middle frontal gyrus | 32698.61  ±7396.35 | 32618.01  ±7039.02 | 34329.44  ±6982.98 | 32832.66  ±7498.28 | 33659.43  ±7900.48 | 29561.39  ±6697.95 | 29319.83  ±5878.72 | 30141.56  ±6135.71 | 32598.06  ±7834.58 |
| Middle occipital gyrus | 9818.41  ±2041.93 | 10050.65  ±1928.63 | 10191.37  ±2023.36 | 9637.02  ±1950.96 | 10234.85  ±2124.62 | 8805.04  ±1727.09 | 8642.49  ±1598.79 | 9103.01  ±1809.1 | 8868.13  ±1765.97 |
| Medial orbital gyrus | 8421.03  ±1636.9 | 8579.41  ±1702.48 | 8609.5  ±1508.53 | 8623.16  ±1725.36 | 8885.61  ±1888.09 | 7760.73  ±1523.52 | 8334.62  ±1566.45 | 8409.12  ±1541.1 | 8578.69  ±1599.66 |
| Postcentral gyrus medial segment | 965.03  ±271.35 | 1030.32  ±245.29 | 1208.35  ±273.46 | 988.73  ±268.32 | 1040.17  ±290.77 | 1031.03  ±294.45 | 849.79  ±203.96 | 913.5  ±223.08 | 1105.78  ±309.47 |
| Precentral gyrus medial segment | 5234.81  ±1129.07 | 5647.8  ±1242.08 | 5998.68  ±1371.74 | 5254.33  ±1268.45 | 5535.35  ±1270.37 | 4954.72  ±1135.67 | 4602.7  ±1072.54 | 4909.44  ±1020.28 | 5275.73  ±1070.65 |
| Superior frontal gyrus medial segment | 11983.96  ±2544.54 | 11593.45  ±2429.51 | 13107.92  ±2781.97 | 11733.73  ±2585.11 | 11897.65  ±2799.34 | 11565.87  ±2728.43 | 10514.4  ±2039.92 | 11086.04  ±2056.95 | 12756.12  ±3356.81 |
| Middle temporal gyrus | 28345.73  ±5529.23 | 29044.43  ±5335.15 | 30384.05  ±5881.83 | 28338.61  ±5694.67 | 29436.66  ±6645.41 | 25489.51  ±5664.63 | 26059.64  ±4875.59 | 26522.59  ±4825.76 | 26942.36  ±5882.76 |
| Occipital pole | 2958.72  ±812.5 | 3049.8  ±790.35 | 3393.14  ±872.09 | 2792.43  ±784.89 | 2971.49  ±763.37 | 2938.18  ±671.43 | 2432.27  ±662.28 | 2531.81  ±584.3 | 2702.47  ±712.78 |
| Occipital fusiform gyrus | 7913.78  ±1486.54 | 8296.14  ±1747.86 | 8473.22  ±1655.29 | 7887.1  ±1626.02 | 8355.77  ±1870.59 | 7416.91  ±1556.55 | 7059.34  ±1467.42 | 7473.4  ±1326.9 | 7717.34  ±1599.86 |
| Opercular part of the inferior frontal gyrus | 6263.19  ±1333.46 | 6332.38  ±1336.79 | 6724.1  ±1154.22 | 6218.84  ±1348.1 | 6639.71  ±1634.89 | 5620.94  ±1369.09 | 5642.38  ±1073.21 | 5757.75  ±1366.92 | 5870.24  ±1372.8 |
| Orbital part of the inferior frontal gyrus | 2780.81  ±607.24 | 2888.43  ±607.16 | 2966.26  ±707.28 | 2865.11  ±685.49 | 3003.83  ±735.87 | 2607.8  ±617.77 | 2554.42  ±566.94 | 2688.52  ±613.26 | 2904.63  ±672.85 |
| Posterior cingulate gyrus | 9326.81  ±2177.61 | 9899.58  ±1962.58 | 10158.19  ±2083.56 | 9576.85  ±2024.68 | 10199.75  ±2298.33 | 8823.29  ±2277.97 | 9231.74  ±2016.21 | 9639.37  ±1947.21 | 9190.69  ±2051.2 |
| Precuneus | 19205.01  ±3921.75 | 20282.97  ±4153.34 | 21814.72  ±4313.7 | 19406.8  ±4277.88 | 20687.07  ±4828.07 | 18483.6  ±4486.18 | 17660.72  ±3872.67 | 18652.9  ±3788.93 | 19409.05  ±4202.48 |
| Parahippocampal gyrus | 6149.32  ±1203.3 | 6688.05  ±1307.03 | 6649.23  ±1279.88 | 6207.15  ±1229.97 | 6865.67  ±1469.09 | 5898.1  ±1169.21 | 6090.34  ±1286.49 | 6632.54  ±1204.79 | 6670.93  ±1493.63 |
| Posterior insula | 3680.32  ±692.43 | 3928  ±828.91 | 3792.1  ±856.39 | 3619.77  ±764.92 | 3883.77  ±826.59 | 3144.55  ±702.96 | 3198.92  ±632.26 | 3318.36  ±708.73 | 3008.5  ±701.67 |
| Parietal operculum | 5035  ±1115.85 | 5235.14  ±1174.08 | 5685.26  ±1342.33 | 5154.14  ±1261.22 | 5341.57  ±1296.8 | 4741.41  ±1290.47 | 4674.25  ±1075.76 | 4721.45  ±1062.94 | 4887.89  ±1218.72 |
| Postcentral gyrus | 17367.34  ±3716.22 | 17854.01  ±3426.63 | 19880.89  ±3374.29 | 17006.63  ±3574.61 | 18317.9  ±3729.48 | 17413.15  ±3397.91 | 15059.71  ±2758.37 | 15849.72  ±3071.43 | 17428.58  ±3772.47 |
| Posterior orbital gyrus | 5018  ±1145.65 | 5294.65  ±1118.95 | 5312.93  ±1149.22 | 5052.62  ±1173.85 | 5539.87  ±1281.96 | 4884.04  ±1006.04 | 4631.78  ±1004.58 | 5022.76  ±997.58 | 5070.42  ±912.8 |
| Planum polare | 4390.97  ±891.55 | 4551.59  ±923.98 | 4682.7  ±957.67 | 4428.55  ±913.67 | 4666.35  ±998.69 | 3987.22  ±819.35 | 4153.98  ±789.36 | 4340.87  ±851.58 | 4325.8  ±964.39 |
| Precentral gyrus | 22891.2  ±5072 | 23028.64  ±4454.08 | 25716.97  ±4748.67 | 21864.85  ±4721.47 | 23357.2  ±4949.97 | 21592.3  ±4417.44 | 19324.8  ±3714.13 | 20250.23  ±3991.12 | 22444.08  ±4859.79 |
| Planum temporale | 4215.53  ±960.49 | 4518.31  ±1062.77 | 4942.04  ±1434.26 | 4307.02  ±1069.19 | 4623.66  ±1228.84 | 4028.28  ±970.86 | 3798.15  ±809.44 | 3957.94  ±898.67 | 4142.5  ±890.29 |
| Subcallosal area | 1888.93  ±419.62 | 2038.13  ±500.08 | 1992.14  ±526.5 | 1932.24  ±470.46 | 2097.25  ±533.03 | 1725.22  ±406.22 | 1864.67  ±497.75 | 1967.55  ±429.55 | 1926.2  ±437.99 |
| Superior frontal gyrus | 27279.59  ±5610.24 | 27275.71  ±5709.43 | 29956.43  ±6211.79 | 27386.7  ±5917.34 | 28202.96  ±6069 | 26066.67  ±5416.85 | 24952.6  ±4594.83 | 26057.71  ±4933.86 | 29229.36  ±6342.21 |
| Supplementary motor cortex | 9975.13  ±2236.19 | 10180.46  ±2133.71 | 11282.94  ±2173.19 | 9988.21  ±2277.48 | 10179.65  ±2406.57 | 9793.95  ±2297.85 | 8806.13  ±1750.05 | 9488.94  ±1901.84 | 10266.2  ±2270.36 |
| Supramarginal gyrus | 14592.65  ±2820.18 | 15354.76  ±3183.9 | 16090.11  ±2900.92 | 14432.39  ±3052.38 | 15232.73  ±3246.01 | 13428.67  ±2872.69 | 12902.03  ±2391.3 | 13909.6  ±2734.24 | 14271.25  ±3239.37 |
| Superior occipital gyrus | 6973.36  ±1481.21 | 7424.42  ±1428.78 | 7487.04  ±1212.13 | 6752.66  ±1413.19 | 7241.63  ±1587.01 | 6405.72  ±1087.24 | 5787.75  ±1129.02 | 6289.25  ±1282.27 | 6128.68  ±1344.21 |
| Superior parietal lobule | 16387.72  ±3283.47 | 16673.99  ±3347.11 | 18515.86  ±3345.74 | 16047.91  ±3398.59 | 16998.65  ±3694.74 | 15481.84  ±3023.26 | 14858.2  ±2652.95 | 15606.33  ±2987.75 | 16368.87  ±3295.24 |
| Superior temporal gyrus | 14287.73  ±2728.55 | 14809.71  ±2835.58 | 14863.62  ±3150.36 | 14412.7  ±3083.13 | 14957.12  ±3406.45 | 12752.85  ±2894.55 | 12869.41  ±2294.87 | 13320.57  ±2630.6 | 13289.89  ±2592.94 |
| Temporal pole | 12973.32  ±2796.13 | 14049.87  ±3188.86 | 15234.02  ±3264.79 | 12991.36  ±3134.23 | 14213.28  ±3448.7 | 13120.77  ±3137.65 | 12288.03  ±2380.45 | 13328.03  ±2630.34 | 13840.58  ±3123.12 |
| Triangular part of the inferior frontal gyrus | 7048.26  ±1414.14 | 6997.65  ±1481.61 | 7084.78  ±1389.25 | 7097.35  ±1467.87 | 7261.87  ±1709.38 | 6237.07  ±1356.48 | 6314.26  ±1317.04 | 6384.53  ±1167.26 | 6536.83  ±1300.56 |
| Transverse temporal gyrus | 2672.74  ±681.34 | 2741.29  ±637.36 | 2912.47  ±694.84 | 2662.69  ±720.9 | 2804.77  ±697.62 | 2355.24  ±613.68 | 2328.65  ±589.23 | 2353.08  ±642.85 | 2252.76  ±521.85 |

Raw regional volumes (units: mL). Acronyms used: HC = healthy control, PRE = PreHD, HD = manifest HD , P = PREDICT, T = TRACK, I = IMAGE. Standard errors are shown.

Supplementary Table 7: Adjusted Regional Volumes

| Biomarker | PRE_P | PRE_T | PRE_I | HD_P | HD_T | HD_I |
| --- | --- | --- | --- | --- | --- | --- |
| Non-ventricular CSF | 14595.14  ±3390.96 | 14742.98  ±2543.23 | 11747.66  ±5785.07 | 19799.83  ±3575.74 | 30045.3  ±2670.54 | 27534.43  ±6593.51 |
| 3rd Ventricle | 82.38  ±55.89 | 166.19  ±53.68 | 134.12  ±118.76 | 132.82  ±58.73 | 532.37  ±56.37 | 536.2  ±135.54 |
| 4th Ventricle | -85.09  ±76.29 | 10.79  ±69.98 | 360.96  ±148.82 | -92  ±78.08 | 192.69  ±73.49 | 252.07  ±169.63 |
| 5th Ventricle | -0.08  ±0.34 | 0.06  ±0.33 | -0.53  ±0.69 | -0.22  ±0.37 | -1.1  ±0.34 | -1.93  ±0.79 |
| Accumbens Area | -12.02  ±7.35 | -31.68  ±6.08 | -38.63  ±13.12 | -13.36  ±8.49 | -46.05  ±6.38 | -64.81  ±14.95 |
| Amygdala | -92.83  ±26.7 | -139.18  ±25.48 | -206.57  ±59.2 | -129.84  ±30.18 | -303.98  ±26.76 | -338.87  ±67.49 |
| Pons | -345.01  ±139.63 | -273.76  ±140.25 | -317.88  ±313.97 | -442.97  ±143.37 | -668.97  ±147.27 | -405.07  ±357.82 |
| Brain Stem | -229.61  ±83.61 | -199.08  ±81.82 | -70.67  ±162.13 | -320.84  ±87.33 | -424.69  ±85.94 | -239.46  ±184.75 |
| Caudate | -385.98  ±57.87 | -382.32  ±42.56 | -176.49  ±109.83 | -492.41  ±65.31 | -799.23  ±44.69 | -561.8  ±125.16 |
| Cerebellum Exterior | 1830.33  ±968.83 | -603.77  ±955.42 | 2815.68  ±1734.62 | 1921.02  ±1039.22 | 491.87  ±1003.38 | 5853.27  ±1976.45 |
| Cerebellum White Matter | 42.03  ±505.76 | -401.45  ±378.71 | -1465.16  ±1305.74 | 282.08  ±644.43 | 452.81  ±397.74 | 1169.98  ±1488.75 |
| Cerebral Exterior | 2.14  ±0.73 | 2.21  ±0.82 | 1.55  ±1.7 | 2.05  ±0.86 | 5.66  ±0.86 | 7.23  ±1.94 |
| 3rd Ventricle (Posterior part) | 101.39  ±36.01 | 111.02  ±39.71 | 98.96  ±74.59 | 121.78  ±37.74 | 266.43  ±41.7 | 497.95  ±85.01 |
| Hippocampus | -161.91  ±60.03 | -167.3  ±56.06 | -76.42  ±142.81 | -225.98  ±65.39 | -339.61  ±58.88 | -507.39  ±162.76 |
| Inf Lat Vent | 58.33  ±41.11 | 62.1  ±49.31 | -54.97  ±86.13 | 63.65  ±44.24 | 227.03  ±51.79 | -2  ±98.34 |
| Lateral Ventricle | -228.47  ±1180.82 | 482.07  ±1159.46 | 301.12  ±2955.41 | 762.71  ±1230.54 | 3163.14  ±1217.16 | 1192.28  ±3379.01 |
| Pallidum | -268.76  ±37.76 | -352.49  ±30.48 | -212.54  ±74.22 | -306.54  ±40.51 | -637.13  ±32.01 | -553.52  ±84.58 |
| Putamen | -355.34  ±62.13 | -430.59  ±50.99 | -285.46  ±139.91 | -392.43  ±70.22 | -620.49  ±53.55 | -588.29  ±159.63 |
| Thalamus Proper | -197.22  ±84.91 | -256.01  ±68.51 | -50.02  ±155.02 | -160.02  ±116.55 | -276.78  ±71.94 | -194.57  ±176.91 |
| Ventral DC | -100.57  ±55.78 | -140.13  ±49.61 | 188.11  ±134.69 | -148.85  ±65.45 | -257.23  ±52.09 | -147.17  ±153.59 |
| Ventricular Lining | 92.01  ±82.74 | 40.68  ±102.04 | -19.74  ±274.5 | 243.2  ±96.22 | 648.27  ±107.08 | 380.63  ±313.43 |
| Optic Chiasm | 7.81  ±2.08 | 8.69  ±2.27 | 12.09  ±4.85 | 13.65  ±2.61 | 27.99  ±2.39 | 33.94  ±5.53 |
| Cerebellar Vermal Lobules I-V | 116.61  ±58.05 | 121.35  ±58.74 | -97.6  ±118.11 | 105.09  ±61.37 | 192.32  ±61.69 | 27.14  ±134.57 |
| Cerebellar Vermal Lobules VI-VII | 1.34  ±24.43 | -15.33  ±23.32 | 39.26  ±49.93 | 13.03  ±26 | 32.54  ±24.49 | 171.04  ±56.89 |
| Cerebellar Vermal Lobules VIII-X | 35.55  ±37.73 | -28.33  ±36.01 | 83.49  ±74.85 | 44.42  ±39.81 | 42.7  ±37.82 | 146.89  ±85.27 |
| Basal Forebrain | -46.18  ±11.45 | -41.86  ±9.84 | -30.69  ±21.94 | -53.81  ±13.51 | -100.86  ±10.33 | -75.65  ±25.01 |
| Temporal White Matter | -1233  ±536.52 | -1383.6  ±502.71 | -1692.23  ±1231.26 | -1982.34  ±601.25 | -3027.65  ±527.91 | -4391.42  ±1402.55 |
| Insula White Matter | -1692.14  ±264.79 | -1638.97  ±207.04 | -943.19  ±541.55 | -2092.27  ±283.21 | -3010.79  ±217.43 | -3262.25  ±617.15 |
| Cingulate White Matter | -60.71  ±95.94 | -379.82  ±94.3 | -269.3  ±167.42 | -135.86  ±103.74 | -610.67  ±99.04 | -514.9  ±190.93 |
| Frontal White Matter | -3876.62  ±1259.03 | -2276.81  ±1055.89 | -1272.88  ±2511.11 | -5930.76  ±1464.66 | -6889.38  ±1108.88 | -10399.6  ±2861.41 |
| Occipital White Matter | -1230.46  ±622.43 | -1665.19  ±484.72 | -751.88  ±1140.92 | -2110.72  ±724.45 | -2355.04  ±509.06 | -2126.6  ±1300.49 |
| Parietal White Matter | -1393.89  ±877.5 | -1557.78  ±773.01 | -1302.67  ±1938.04 | -2900.2  ±985.85 | -5447.79  ±811.79 | -7404.23  ±2208.65 |
| Corpus Callosum | -385.76  ±212.54 | -543.62  ±215.87 | -476.19  ±503.16 | -595.16  ±218.52 | -1346.03  ±226.69 | -1396.92  ±573.51 |
| Claustrum | 22.78  ±47.52 | -68.83  ±45.3 | -154.73  ±113.08 | -7.4  ±51.71 | -156.11  ±47.58 | -244.25  ±128.86 |
| Anterior cingulate gyrus | -269.99  ±125.25 | -123.52  ±112.49 | -115.91  ±224.81 | -308.65  ±131.14 | 22.86  ±118.14 | -109.95  ±256.21 |
| Anterior insula | -139.09  ±78.07 | -189.58  ±80.11 | -202.73  ±166.34 | -281.74  ±84.42 | -647.72  ±84.14 | -352.54  ±189.67 |
| Anterior orbital gyrus | -37.57  ±40.44 | 4.83  ±35.28 | 4.68  ±78.19 | -49.06  ±47.4 | 6.19  ±37.05 | 128.63  ±89.1 |
| Angular gyrus | -296.63  ±231.87 | -419.54  ±186.95 | -80.95  ±402.18 | -457.48  ±248.81 | -854.39  ±196.33 | -277.77  ±458.4 |
| Calcarine cortex | -326.89  ±146.07 | -331.1  ±156.28 | -189.46  ±361.1 | -431.26  ±152.16 | -1039.48  ±164.13 | -1046.23  ±411.59 |
| Central operculum | -125.85  ±80.52 | 115.57  ±84.32 | -250.47  ±163.02 | -216.21  ±85.42 | -228.39  ±88.55 | -181.42  ±185.78 |
| Cuneus | -468.54  ±149.78 | -343.2  ±129.35 | -529.64  ±331.73 | -645.75  ±158.22 | -1009.53  ±135.87 | -1378.05  ±378.05 |
| Entorhinal area | -85.63  ±48.31 | -70.13  ±48.42 | -94.14  ±104.66 | -94.34  ±53.27 | -142.09  ±50.85 | -123.69  ±119.27 |
| Frontal operculum | -44.78  ±53.49 | -41.3  ±51.95 | -69.95  ±100.37 | -97.4  ±55.19 | -189.32  ±54.56 | -169.74  ±114.4 |
| Frontal pole | -82.93  ±96.77 | 17.17  ±54.43 | 270.75  ±177.86 | -252.8  ±122.46 | -127.61  ±57.17 | 352.52  ±202.87 |
| Fusiform gyrus | -107.05  ±151.79 | -134.16  ±145.66 | -159.24  ±265.86 | -151.04  ±163.98 | -88.84  ±152.98 | -123.99  ±302.97 |
| Gyrus rectus | 22.01  ±50.39 | -16.23  ±47.76 | -35.53  ±94.89 | 6.6  ±56.78 | 12.69  ±50.16 | 157.8  ±108.13 |
| Inferior occipital gyrus | -339.03  ±148.09 | -256.33  ±130.51 | -526.45  ±294.52 | -492.59  ±173.97 | -918.56  ±137.07 | -1056.85  ±335.66 |
| Inferior temporal gyrus | -142.86  ±190.51 | 5.54  ±173.29 | -842.79  ±354.4 | -227.84  ±204.32 | -280.39  ±181.99 | -788.85  ±403.87 |
| Lingual gyrus | -182.41  ±148.57 | -326.64  ±159.3 | -97.88  ±371.44 | -333.44  ±164.35 | -971.81  ±167.3 | -1368.68  ±423.54 |
| Lateral orbital gyrus | 14.23  ±40.87 | 42.11  ±37.13 | 103.81  ±75.37 | -7.79  ±47.42 | -53.35  ±39 | 176.37  ±85.9 |
| Middle cingulate gyrus | 60.81  ±93.41 | -201.69  ±88.05 | -522.92  ±151.77 | 40.07  ±101.28 | 88.43  ±92.47 | -238.53  ±172.93 |
| Medial frontal cortex | -83.89  ±51.19 | -70.41  ±49.66 | 72.37  ±106.07 | -119.02  ±57.39 | -102.66  ±52.16 | 326.91  ±120.9 |
| Middle frontal gyrus | -883.93  ±397.02 | -38.54  ±317.52 | -244.11  ±696.98 | -1321.73  ±427.17 | -907.32  ±333.45 | -158.6  ±794.64 |
| Middle occipital gyrus | -231.57  ±126.81 | -90.07  ±108.88 | -197.72  ±213.62 | -386.18  ±148.69 | -523.81  ±114.34 | -437.85  ±243.43 |
| Medial orbital gyrus | 96.37  ±78.23 | 116.33  ±67.08 | 121.35  ±129.05 | 133.97  ±90.17 | 138.4  ±70.45 | 290.27  ±147.08 |
| Postcentral gyrus medial segment | 9.95  ±30.56 | -12.06  ±22.88 | -48.93  ±63.19 | 12  ±32.6 | -94.34  ±24.03 | -104.33  ±72.01 |
| Precentral gyrus medial segment | -211.74  ±89.67 | -275.83  ±77.76 | -322.68  ±164.65 | -312.67  ±97.02 | -526.32  ±81.68 | -548.5  ±187.62 |
| Superior frontal gyrus medial segment | -330.54  ±137.5 | -149.57  ±113.59 | 323.93  ±264.62 | -510.9  ±152.19 | -76.51  ±119.29 | 477.95  ±301.55 |
| Middle temporal gyrus | -507.24  ±269.2 | -547.1  ±242.72 | -1059.75  ±560.68 | -715.1  ±281.27 | -1303.07  ±254.89 | -1605.16  ±639.13 |
| Occipital pole | -161.91  ±64.57 | -176.05  ±49.42 | -57.23  ±148.3 | -214.13  ±83.98 | -388.02  ±51.9 | -577.21  ±168.96 |
| Occipital fusiform gyrus | -154.51  ±90.46 | -178.81  ±86.51 | -79.46  ±173.24 | -272.49  ±102.19 | -487.68  ±90.85 | -396.58  ±197.39 |
| Opercular part of the inferior frontal gyrus | -120.13  ±98.18 | 43.65  ±101.54 | -375.17  ±193.03 | -288.09  ±103.91 | -261.92  ±106.63 | -486.39  ±220 |
| Orbital part of the inferior frontal gyrus | 37.42  ±47.99 | 4.28  ±49.61 | 61.27  ±99.68 | 19.08  ±50.85 | -98.15  ±52.11 | 89.93  ±113.64 |
| Posterior cingulate gyrus | 149.09  ±107.67 | 21.45  ±90.42 | -42.9  ±219.34 | 93.5  ±112.86 | 83.42  ±94.96 | -216.63  ±250.06 |
| Precuneus | -91.52  ±231 | -197.07  ±201.39 | -627.28  ±458.98 | -339.85  ±247.27 | -856.29  ±211.5 | -1256.36  ±523.12 |
| Parahippocampal gyrus | 13.6  ±69.59 | 46.05  ±61.51 | -2.35  ±127.28 | 72.12  ±78.75 | 131.79  ±64.6 | -41.3  ±145.1 |
| Posterior insula | -152.37  ±43.77 | -152.89  ±39.45 | -180.99  ±91.66 | -248.39  ±47.99 | -421.1  ±41.43 | -457.09  ±104.44 |
| Parietal operculum | 30.65  ±77.73 | -65.59  ±76.91 | -180.77  ±162 | -34.67  ±80.67 | -347.66  ±80.77 | -385.52  ±184.62 |
| Postcentral gyrus | -475.18  ±213.99 | -17.68  ±182.24 | -179.98  ±383.78 | -627.48  ±242.08 | -1298.24  ±191.41 | -1649.79  ±437.42 |
| Posterior orbital gyrus | -118.69  ±79.4 | 89.26  ±70.98 | 202.61  ±149.08 | -153.56  ±83.24 | -102.7  ±74.54 | -37.76  ±170.09 |
| Planum polare | -37.19  ±45.45 | -22  ±45.02 | -119.45  ±76.76 | -70.51  ±48.22 | -36.93  ±47.28 | -95.22  ±87.47 |
| Precentral gyrus | -1359.56  ±265 | -303.26  ±221.8 | -1035.43  ±414.11 | -1640.9  ±294.67 | -1592  ±232.91 | -1927.49  ±472.03 |
| Planum temporale | 5.96  ±67.62 | -56  ±67.36 | -260.17  ±170.86 | -62.15  ±71.66 | -374.01  ±70.75 | -444.62  ±194.76 |
| Subcallosal area | 38.38  ±32.42 | 13.99  ±29.36 | 26.16  ±67.83 | 51  ±37.95 | 2.39  ±30.83 | 107.13  ±77.29 |
| Superior frontal gyrus | -190.12  ±298.44 | 76.32  ±257.26 | 52.61  ±529.19 | -418  ±348.52 | -86.1  ±270.19 | 1053.27  ±603.37 |
| Supplementary motor cortex | -244.5  ±120.41 | -381.83  ±99.5 | -95.81  ±218.17 | -405.01  ±135.83 | -314.88  ±104.49 | -564.84  ±248.62 |
| Supramarginal gyrus | -384.24  ±179.98 | -574.51  ±170.55 | -793.02  ±367.73 | -564.71  ±196.11 | -804.71  ±179.11 | -1097.9  ±419.41 |
| Superior occipital gyrus | -283.75  ±117.37 | -373.06  ±101.2 | -329.2  ±224.56 | -429.4  ±139.29 | -849.84  ±106.3 | -955.96  ±255.91 |
| Superior parietal lobule | -479.37  ±231.44 | -84.69  ±187.38 | -963.8  ±376.99 | -675.41  ±253.6 | -404.62  ±196.79 | -1082.22  ±429.59 |
| Superior temporal gyrus | -153.9  ±145.53 | -353.51  ±149.09 | -325.26  ±377.16 | -353.73  ±154.85 | -895.74  ±156.57 | -560.25  ±430.03 |
| Temporal pole | -58.55  ±196.59 | -148.76  ±197.24 | -381.03  ±408.38 | -82.23  ±209.01 | -359.71  ±207.14 | -1023.85  ±465.39 |
| Triangular part of the inferior frontal gyrus | -118.94  ±91.39 | -2.03  ±90.78 | -32.76  ±158.42 | -242.96  ±100.33 | -243.19  ±95.35 | -216.84  ±180.55 |
| Transverse temporal gyrus | -117.97  ±50.8 | -36.22  ±45.98 | -229.69  ±94.98 | -192.79  ±53.1 | -255.52  ±48.28 | -496.14  ±108.32 |

Adjusted regional volumes (units: mL). Acronyms used: HC = healthy control, PRE = PreHD, HD = manifest HD , P = PREDICT, T = TRACK, I = IMAGE. Standard errors are shown.

Supplementary Table 8: Effect Size By Regional Volume

| Biomarker | PREDICT-HD | TRACK-HD | IMAGE-HD |
| --- | --- | --- | --- |
| Non-ventricular CSF | 0.71 | 0.89 | 0.62 |
| 3rd Ventricle | 0.21 | 0.42 | 0.52 |
| 4th Ventricle | -0.17 | 0.05 | 0.96 |
| 5th Ventricle | -0.03 | 0.06 | -0.2 |
| Accumbens Area | -0.25 | -0.85 | -0.96 |
| Amygdala | -0.57 | -0.76 | -1.02 |
| Pons | -0.39 | -0.36 | -0.3 |
| Brain Stem | -0.46 | -0.36 | -0.23 |
| Caudate | -1.12 | -1.44 | -0.47 |
| Cerebellum Exterior | 0.27 | -0.09 | 0.62 |
| Cerebellum White Matter | <0.01 | -0.19 | -0.45 |
| Cerebral Exterior | 0.51 | 0.41 | 0.15 |
| 3rd Ventricle (Posterior part) | 0.48 | 0.39 | 0.3 |
| Hippocampus | -0.44 | -0.43 | -0.12 |
| Inf Lat Vent | 0.22 | 0.18 | -0.19 |
| Lateral Ventricle | -0.07 | 0.08 | 0.24 |
| Pallidum | -1.07 | -1.76 | -0.78 |
| Putamen | -0.93 | -1.35 | -0.57 |
| Thalamus Proper | -0.48 | -0.61 | -0.12 |
| Ventral DC | -0.32 | -0.44 | 0.38 |
| Ventricular Lining | 0.22 | 0.14 | -0.03 |
| Optic Chiasm | 0.68 | 0.65 | 0.91 |
| Cerebellar Vermal Lobules I-V | 0.31 | 0.29 | -0.19 |
| Cerebellar Vermal Lobules VI-VII | 0.02 | -0.09 | 0.29 |
| Cerebellar Vermal Lobules VIII-X | 0.13 | -0.11 | 0.26 |
| Basal Forebrain | -0.67 | -0.66 | -0.4 |
| Temporal White Matter | -0.38 | -0.34 | -0.44 |
| Insula White Matter | -1.01 | -1.23 | -0.49 |
| Cingulate White Matter | -0.1 | -0.56 | -0.53 |
| Frontal White Matter | -0.51 | -0.31 | -0.14 |
| Occipital White Matter | -0.32 | -0.56 | -0.13 |
| Parietal White Matter | -0.28 | -0.23 | -0.04 |
| Corpus Callosum | -0.28 | -0.31 | -0.21 |
| Claustrum | 0.09 | -0.28 | -0.37 |
| Anterior cingulate gyrus | -0.33 | -0.19 | -0.16 |
| Anterior insula | -0.28 | -0.42 | -0.34 |
| Anterior orbital gyrus | -0.16 | <0.01 | 0.07 |
| Angular gyrus | -0.2 | -0.3 | -0.16 |
| Calcarine cortex | -0.33 | -0.33 | -0.19 |
| Central operculum | -0.23 | 0.19 | -0.39 |
| Cuneus | -0.46 | -0.4 | -0.55 |
| Entorhinal area | -0.3 | -0.21 | -0.33 |
| Frontal operculum | -0.13 | -0.17 | -0.14 |
| Frontal pole | -0.14 | 0.04 | 0.47 |
| Fusiform gyrus | -0.12 | -0.1 | -0.11 |
| Gyrus rectus | 0.06 | -0.08 | -0.15 |
| Inferior occipital gyrus | -0.38 | -0.33 | -0.53 |
| Inferior temporal gyrus | -0.13 | 0.01 | -0.75 |
| Lingual gyrus | -0.19 | -0.3 | -0.11 |
| Lateral orbital gyrus | 0.07 | 0.15 | 0.62 |
| Middle cingulate gyrus | 0.11 | -0.36 | -1.15 |
| Medial frontal cortex | -0.23 | -0.29 | 0.13 |
| Middle frontal gyrus | -0.34 | -0.05 | -0.16 |
| Middle occipital gyrus | -0.33 | -0.13 | -0.3 |
| Medial orbital gyrus | 0.2 | 0.24 | 0.36 |
| Postcentral gyrus medial segment | 0.06 | -0.1 | -0.27 |
| Precentral gyrus medial segment | -0.39 | -0.55 | -0.56 |
| Superior frontal gyrus medial segment | -0.37 | -0.21 | 0.36 |
| Middle temporal gyrus | -0.29 | -0.32 | -0.55 |
| Occipital pole | -0.47 | -0.5 | -0.08 |
| Occipital fusiform gyrus | -0.28 | -0.31 | -0.11 |
| Opercular part of the inferior frontal gyrus | -0.23 | 0.03 | -0.61 |
| Orbital part of the inferior frontal gyrus | 0.14 | <0.01 | 0.22 |
| Posterior cingulate gyrus | 0.21 | 0.06 | 0.03 |
| Precuneus | -0.06 | -0.13 | -0.36 |
| Parahippocampal gyrus | 0.03 | 0.12 | 0.01 |
| Posterior insula | -0.6 | -0.65 | -0.65 |
| Parietal operculum | 0.07 | -0.11 | -0.31 |
| Postcentral gyrus | -0.37 | 0.01 | -0.18 |
| Posterior orbital gyrus | -0.23 | 0.18 | 0.49 |
| Planum polare | -0.13 | -0.07 | -0.57 |
| Precentral gyrus | -0.87 | -0.21 | -0.85 |
| Planum temporale | <0.01 | -0.11 | -0.39 |
| Subcallosal area | 0.21 | 0.05 | 0.11 |
| Superior frontal gyrus | -0.13 | -0.02 | 0.08 |
| Supplementary motor cortex | -0.33 | -0.59 | -0.06 |
| Supramarginal gyrus | -0.32 | -0.48 | -0.65 |
| Superior occipital gyrus | -0.4 | -0.57 | -0.58 |
| Superior parietal lobule | -0.37 | -0.09 | -0.83 |
| Superior temporal gyrus | -0.16 | -0.36 | -0.25 |
| Temporal pole | -0.03 | -0.12 | -0.34 |
| Triangular part of the inferior frontal gyrus | -0.19 | -0.05 | -0.01 |
| Transverse temporal gyrus | -0.38 | -0.13 | -0.68 |

Standardised effect size between healthy control and PreHD groups, for each regional volume in the PREDICT 1.5T+3T, TRACK 3T and IMAGE 3T cohorts.
